# Production of aromatic amino acids and their derivatives by *Escherichia coli* and *Corynebacterium glutamicum*

**DOI:** 10.1007/s11274-025-04264-3

**Published:** 2025-02-07

**Authors:** Takashi Hirasawa, Yasuharu Satoh, Daisuke Koma

**Affiliations:** 1 School of Life Science and Technology, Institute of Science Tokyo, 4259 Nagatsuta-cho, Midori-ku, Yokohama, Kanagawa 226-8501 Japan; 2 Faculty of Engineering, Hokkaido University, N13 & W8, Kita-ku, Sapporo, Hokkaido 060-8628 Japan; 3 Osaka Research Institute of Industrial Science and Technology, 1-6-50 Morinomiya, Joto-ku, Osaka, 536-8553 Japan

**Keywords:** Aromatic amino acids, Aromatic amino acid derivatives, Fermentative production, *Escherichia coli*, *Corynebacterium glutamicum*

## Abstract

Demand for aromatic amino acids (AAAs), such as L-phenylalanine, L-tyrosine, and L-tryptophan, has been increasing as they are used in animal feed and as precursors in the synthesis of industrial and pharmaceutical compounds. These AAAs are biosynthesized through the shikimate pathway in microorganisms and plants, and the reactions in the AAA biosynthesis pathways are strictly regulated at the levels of both gene expression and enzyme activity. Various attempts have been made to produce AAAs and their derivatives using microbial cells and to optimize production. In this review, we summarize the metabolic pathways involved in the biosynthesis of AAAs and their regulation and review recent research on AAA production using industrial bacteria, such as *Escherichia coli* and *Corynebacterium glutamicum*. Studies on fermentative production of AAA derivatives, including L-3,4-dihydroxyphenylalanine, tyrosol, and 3-hydroxytyrosol, are also discussed.

**Supplementary Information:**

The online version contains supplementary material available at 10.1007/s11274-025-04264-3.

## Introduction

Of the 20 standard L-amino acids, four are classified as aromatic amino acids (AAAs); L-phenylalanine (Phe), L-tyrosine (Tyr), L-tryptophan (Trp), and L-Histidine, which have an aromatic ring in their side chain. Three of them, Phe, Trp, and L-histidine, are essential amino acids for humans. Moreover, Phe, Trp, and Tyr are the starting compounds in the biosynthesis of various hormones and neurotransmitters.

Biosynthesis of the AAAs (hereafter, Phe, Trp, and Tyr) in microorganisms and plants has been well studied and is known to be regulated at the levels of transcription and enzyme activity. These AAAs have been used in animal feed and as precursors for the synthesis of industrial and pharmaceutical compounds. Based on knowledge of AAA biosynthetic pathways and their regulation, microbial cells have been engineered for fermentative AAA production. Recently, production of AAA derivatives using microbial cells has also been studied. In this review, we provide an overview of AAA biosynthesis and its regulation in *Escherichia coli* and *Corynebacterium glutamicum*, a coryneform bacterium used as a host for producing amino acids and other materials. Studies on production of AAAs and their derivatives using these bacteria are also introduced. It is known well that *E. coli* exhibits high growth rate and its genetic engineering tools, which can be used for breeding production host species, have highly been developed. As for *C. glutamicum*, various achievements in amino acid production have been conducted. Considering production of AAAs and their derivatives in particular, *C. glutamicum* exhibits higher tolerance to aromatic compounds, such as 4-hydroxybenzoate (Kitade et al. 2018) and *p*-aminobenzoate (Kubota et al. 2016), than other bacteria. Therefore, both *E. coli* and *C. glutamicum* are efficient host species for producing AAAs and their derivatives.

### Biosynthesis of aromatic amino acids

**Fig. 1:**
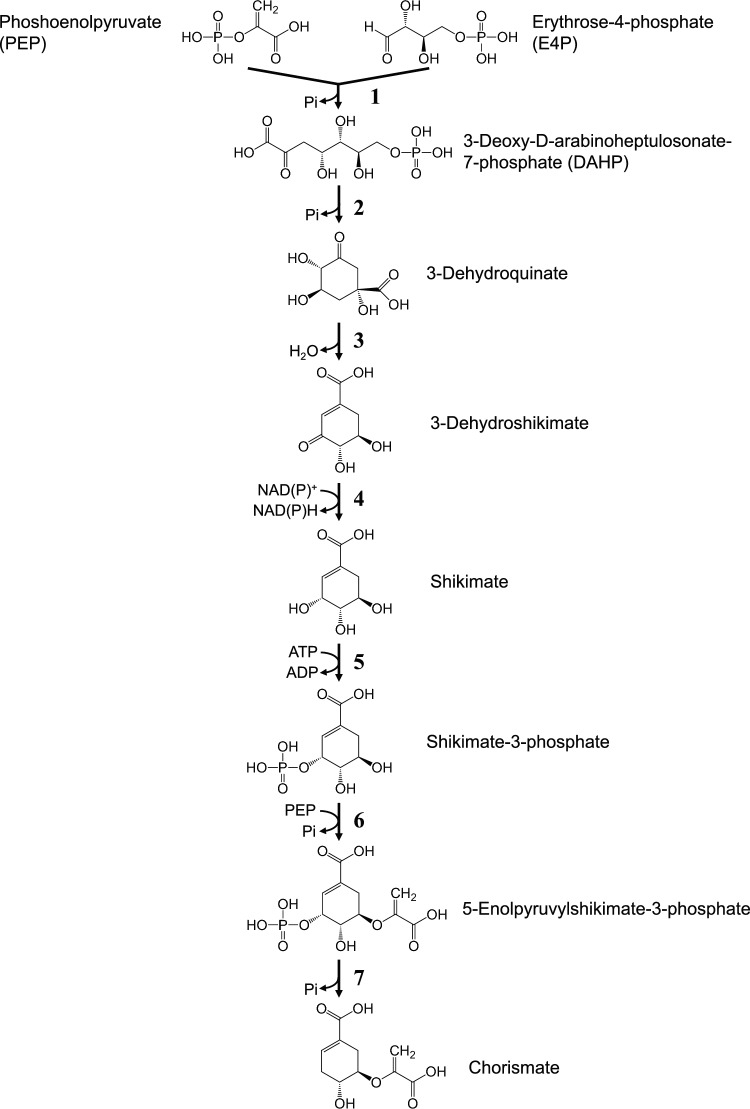
Metabolic pathways for chorismate biosynthesis (the shikimate pathway) in microorganisms

In bacteria, yeasts, fungi, and plants, AAAs are biosynthesized via a common metabolic pathway, the shikimate pathway (Fig. 1). The enzymes, substrates, products, and genes involved in AAA biosynthesis in *E. coli* and *C. glutamicum* are listed in Supplementary Table S1.

**Fig. 2:**
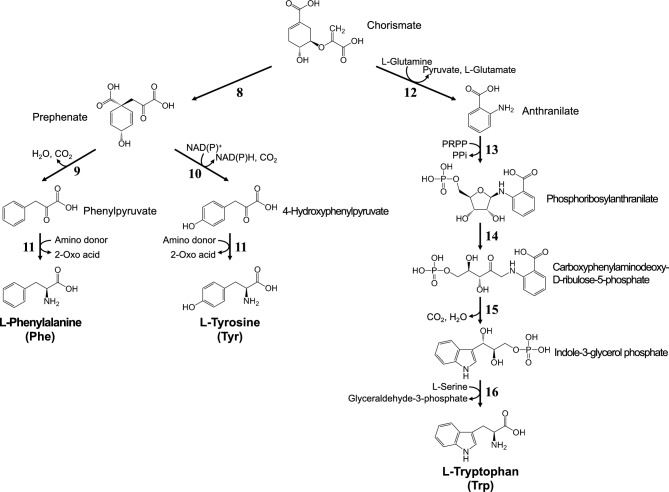
Terminal metabolic pathways for biosynthesis of aromatic amino acids from chorismate in microorganisms

AAAs are biosynthesized from the common metabolite chorismate, which is produced via the shikimate pathway. In this pathway, phosphoenolpyruvate (PEP) and erythrose-4-phosphate (E4P), which are synthesized in the glycolysis and pentose phosphate pathway, respectively, are first condensed to yield 3-deoxy-D-arabinoheptulosonate-7-phosphate (DAHP) in a reaction catalyzed by DAHP synthase (DAHPS) (Reaction 1; the reaction numbers in this section are shown in Figs. 1 and 2 and Supplementary Table S1). Then, DAHP is converted to shikimate via three reactions (Reactions 2–4), and shikimate is converted to chorismate via three enzymatic reactions (Reactions 5–7). In the 5-enolpyruvylshikimate-3-phosphate synthase reaction (Reaction 6), PEP is condensed with shikimate-3-phosphate to yield 5-enolpyruvylshikimate-3-phosphate.

In the terminal pathways for Phe and Tyr biosynthesis (Fig. 2), chorismate is converted to prephenate via Claisen rearrangement with chorismate mutase (CM) (Reaction 8). In Phe biosynthesis, prephenate is metabolized to yield phenylpyruvate via decarboxylation by prephenate dehydratase (PDT) (Reaction 9), whereas in Tyr biosynthesis, chorismate is converted to 4-hydroxyphenylpyruvate via oxidative decarboxylation by prephenate dehydrogenase (PDH) (Reaction 10). Phenylpyruvate and 4-hydroxyphenylpyruvate are then converted to Phe and Tyr, respectively, via transamination by tyrosine aminotransferase (Reaction 11). Some bacteria including *C. glutamicum* and *Pseudomonas aeruginosa* have another Tyr biosynthesis pathway, in which Tyr is biosynthesized from arogenate (pretyrosine), as a conversion product of prephenate (Patel et al. 1977; Fazel and Jensen 1979).

In Trp biosynthesis (Fig. 2), chorismate is converted to anthranilate by anthranilate synthase (Reaction 12). Anthranilate is condensed with phosphoribosylpyrophosphate (PRPP) by anthranilate phosphoribosyltransferase to yield phosphoribosylanthranilate (Reaction 13). Phosphoribosylanthranilate is then metabolized by phosphoribosylanthranilate isomerase to produce carboxyphenylaminodeoxy-D-ribulose-5-phosphate (Reaction 14), which is further converted to indole-3-glycerol phosphate by indole-3-glycerol phosphate synthase (Reaction 15). Finally, indole-3-glycerol phosphate is converted to indole and 3-phosphoglycelaldehyde by tryptophan synthase α chain (TrpA), and then Trp is synthesized from indole and L-serine by tryptophan synthase β chain (TrpB) (Reaction 16).

In relation to the shikimate pathway, *C. glutamicum* possesses metabolic pathway for assimilating quinate and shikimate (Supplementary Fig. S1) (Teramoto et al. 2009; Kubota et al. 2014). It is thought that both quinate and shikimate are incorporated into the *C. glutamicum* cells through a protein encoded by *pcaA*, which belongs to the major facilitator superfamily. Quinate and shikimate are converted to 3-dehydroquinate and 3-dehydroshikimate, respectively, by shikimate 5-dehydrogenase encoded by *qsuD*. 3-Dehydroquinate is converted to 3-dehydroshikimate by 3-dehydroquinate dehydratase encoded by *qsuC*, which belongs to the shikimate pathway. 3-Dehydroshikimate is further metabolized to protocatechuate by 3-dehydroshikimate dehydratase encoded by *qsuB*. It is assumed that protocatechuate is finally metabolized to succinyl-CoA and acetyl-CoA, which are further metabolized in the TCA cycle. Additionally, *qsuA*, *qsuB*, *qsuC*, and *qsuD* genes constitute a single operon on the *C. glutamicum* genome and its expression is regulated by the chorismate-dependent transcriptional regulator encoded by *qsuR* which is located just upstream of the *qsuABCD* operon in the opposite direction (Kubota et al. 2014).

### Regulation of aromatic amino acid biosynthesis in *E. coli* and *C. glutamicum*

#### Regulation of aromatic amino acid biosynthesis by modulating enzyme activity

In the AAA biosynthesis pathway, carbon flow to the shikimate pathway is regulated through feedback inhibition, as the terminal AAAs control the enzymatic activity of DAHPS. *E. coli* has three isozymes of DAHPS, which are encoded by *aroF*, *aroG*, and *aroH*, and their activities are inhibited by Tyr, Phe, and Trp, respectively (Brown 1968). In contrast, *C. glutamicum* harbors two DAHPS isozymes, which are encoded by *aroF* and *aroG*. The activity of AroG is moderately inhibited by Trp, whereas that of AroF is inhibited by Tyr and Phe (Liu et al. 2008). AroG is the dominant enzyme in the shikimate pathway (Liu et al. 2008).

The biosynthesis of AAAs is also controlled by feedback inhibition of the enzymes catalyzing the terminal reactions, i.e. conversion of chorismate to the AAAs. During Phe biosynthesis in *E. coli*, the activity of the bifunctional enzyme CM-PDT, encoded by *pheA*, is inhibited by Phe (Dopheide et al. 1972). In Tyr biosynthesis in *E. coli*, Tyr inhibits activity of the bifunctional enzyme CM-PDH, which is encoded by *tyrA* (Hudson et al. 1983). In Trp biosynthesis in *E. coli*, Trp inhibits the activities of anthranilate synthase and anthranilate phosphoribosyltransferase (Ito and Crawford 1965).

In *C. glutamicum*, AAA biosynthesis is also controlled by feedback inhibition of the enzymes that catalyze the terminal reactions. AroG from *C. glutamicum* forms a complex of its tetramer with a dimer of CM encoded by *csm* (Li et al. 2013; Burschowsky et al. 2018). Complex formation is not required for DAHPS activity but is essential for CM activity. Phe inhibits complex formation between AroG and Csm, resulting in inhibition of CM activity. Phe also inhibits PDT, which is encoded by *pheA*. Like in *E. coli*, in Trp biosynthesis pathway of *C. glutamicum*, Trp inhibits the activities of both anthranilate synthase and anthranilate phosphoribosyltransferase.

#### Transcriptional regulation of aromatic amino acid biosynthesis

The AAA biosynthesis are also transcriptionally regulated by AAA availability. In *E. coli*, production of DAHPS is controlled by transcriptional repression with the AAAs (i.e. feedback repression). Expression of *aroH* and *aroF*, which encode DAHPS isozymes, is negatively regulated by the transcriptional regulators TyrR and TrpR (Muday et al. 1991). These transcriptional regulators with AAAs bind to the regions upstream of target genes encoding DAHPS to repress their expression.

An important finding regarding the regulation of gene expression in the AAA biosynthesis is attenuation of the *trpEDCBA* operon in *E. coli* (Yanofsky 1981). Expression of this operon is regulated by the availability of Trp-charged tRNA, as *trpL* encodes a Trp-containing leader peptide upstream of the operon. Expression of the *pheA* gene encoding CM-PDT, which has the *pheL* leader peptide sequence upstream, is similarly regulated by the availability of Phe-charged tRNA (Zurawski et al. 1978; Gavini and Pulakat 1991). Expression of the *pheST* operon encoding the Phe-tRNA synthetase complex is also similarly regulated by attenuation (Springer et al. 1985).

Transcription attenuation of the *trpEGDCFBA* operon has also been reported in *C. glutamicum* (Neshat et al. 2014). The *trpL* encoding a leader peptide with Trp residues is located upstream of this operon, and transcription of this operon is enhanced by ribosome stalling at Trp codons in the *trpL* mRNA resulting from depletion of Trp-charged tRNA. The *aroR* gene also encodes a leader peptide upstream of the operon containing *aroF*, which encodes DAHPS (Neshat et al. 2014). The AroR leader peptide contains Phe-Tyr-Phe residues, and transcription of the operon containing *aroF* is enhanced by ribosome stalling at Phe and Tyr codons in *aroR* mRNA under Phe-limited conditions. However, Tyr availability does not affect transcription of this operon.

In addition to attenuation due to ribosome stalling, expression of the *trpEGDCFBA* operon is also regulated by the IclR-type transcriptional regulator LtbR (Brune et al. 2007). The *ltbR* gene is located upstream of the *leuCD* operon, which is related to L-leucine biosynthesis, and LtbR negatively regulates the expression of both the *leuCD* operon and the *trpEGDCFBA* operon. The LtbR consensus binding sequence in the –10 region of the promoter was also found in the promoter regions of the *trpL* and *aroG* genes, which encode the leader peptides for the *trpEGDCFBA* operon and DAHPS, respectively.

### Fermentative production of aromatic amino acids by *E. coli *and *C. glutamicum*

In studies conducted in the 1970s, release from feedback inhibition of DAHPS and the enzymes in the terminal pathways for AAA biosynthesis enhanced the production of AAAs using microbial cells. In these studies, *C. glutamicum* mutant strains showing resistant to AAA analogs, which inhibit cell growth by affecting the related biosynthesis reactions, were isolated as AAA production hosts (Hagino and Nakayama 1973, 1974, 1975; Shiio et al. 1984). In the 1990s, AAA-producing strains were rationally created based on the concept of metabolic engineering (Bailey 1991; Stephanopoulos and Vallino 1991). In this section, we describe studies on fermentative production of AAAs based on metabolic engineering of *E. coli* and *C. glutamicum* reported after 2010 (Supplementary Table S2).

#### Phenylalanine

Modification of the shikimate and terminal biosynthesis pathways is one engineering strategy for producing AAAs in *E. coli* and *C. glutamicum*. Another strategy is enhancement of the supply of substrates for DAHPS, PEP and E4P. Liu et al. (2013) demonstrated that overexpression of a truncated *pheA* that has the coding region for the catalytic domain of CM-PDT and a mutant *aroG* encoding feedback-resistant DAHPS enhanced Phe production by *E. coli* wild-type and elevating expression of the *ydiB* and *aroK* genes encoding shikimate dehydrogenase and shikimate kinase, respectively, further improved the productivity. Ding et al. (2016) found that increase in the amount of shikimate kinase and 5-enolpyruvylshikimate-3-phosphate synthase encoded by *aroL* and *aroA*, respectively, enhanced Phe production in *E. coli* based on absolute quantification of the enzymes related to shikimate synthesis and an in vitro system using purified enzymes. Overexpression of the *aroA* gene successfully increased Phe production by the recombinant *E. coli* strain to about 62 g L^–1^ in fed-batch culture. Wu et al. (2019) applied a dynamic regulation strategy to generate Phe-producing strains of *E. coli*. In this study, modified promoters for *tyrP*, whose transcription is upregulated by the transcriptional regulator TyrR in the presence of Phe, were screened and used for dynamic control of the expression of *aroK*, which encodes shikimate kinase, in the previously constructed Phe-producing *E. coli* mutant strain.

Recently, Wang et al. (2024) reported breeding a Phe-producing strain by expressing endogenous and exogenous genes related to the shikimate pathway and Phe biosynthesis from various promoters in a shikimate-producing *E. coli* strain. In addition, they used adaptive laboratory evolution to isolate *E. coli* cells with tolerance to high Phe concentration and found that *marA*, which encodes a transcriptional regulator, was responsible for tolerance to Phe. Integration of enhanced flux for the shikimate and terminal Phe biosynthesis pathway with high Phe tolerance by overexpression of *marA* yielded about 80 g L^–1^ Phe in fed-batch cultivation.

As with Phe production using engineered *E. coli* strains, increased flux of the shikimate and terminal Phe biosynthesis pathways also enhanced Phe production in *C. glutamicum* (Zhang et al. 2013, 2014). Zhang et al. (2015) investigated the effect of Phe biosynthesis gene overexpression on Phe production to identify the key enzymes involved in Phe production. Subsequently, they introduced various expression modules for identified genes encoding key enzymes in the wild-type strain and evaluated their effects on Phe productivity. Phe production was further improved by modifying the phosphotransferase system (PTS), which is responsible for sugar uptake coupled with conversion of PEP to pyruvate, to supply PEP and blocking the production of lactate and acetate.

Recently, Kataoka et al. (2023) conducted stepwise metabolic engineering of *C. glutamicum* for Phe production. They achieved about 8 g L^–1^ Phe production by overexpressing wild-type *aroH* and mutant *pheA* genes from *E. coli* cloned on a plasmid and the *aroE* gene on the genome combined with disruption of *hdpA*, *qsuB*, *qsuD*, *tyrA*, and *ppc* to avoid utilizing intermediate metabolites in the shikimate pathway for other metabolic pathways, reduce competing Tyr production, and enhance the PEP supply to the DAHPS reaction.

Tachikawa et al. (2024) metabolically engineered *C. glutamicum* for Phe production using adaptive laboratory evolution based on long-term repetitive passage cultures to isolate mutants showing resistance to a Phe analog. They found that analog-resistant mutants had the potential to produce both Phe and Tyr. Since the mutants carried mutations in the *aroG* and *pheA* genes, they analyzed AAA production by the wild-type *C. glutamicum* strain overexpressing both mutant *aroG* and *pheA*, which produced about 3 g L^–1^ Phe. Then, Phe production was further improved up to 6 g L^–1^ by disrupting the *aroP* gene, which encodes AAA permease.

#### Tyrosine

As with Phe production, microorganisms were metabolically engineered for Tyr production by modifying the shikimate and terminal AAA biosynthesis pathways. Juminaga et al. (2012) performed proteomic and metabolomic analyses to identify bottlenecks in Tyr production, which revealed that the activity level of the shikimate dehydrogenase YdiB and low expression of the dehydroquinate synthase AroB are bottlenecks in shikimate production. Based on their bottleneck analysis, they employed expression modules of the genes related to the shikimate pathway in *E. coli* and examined Tyr production, which showed that expressing shikimate pathway-related genes as operons on medium copy number plasmids resulted in more than 2 g L^–1^ Tyr production, which is 80% of the theoretical yield. Moreover, modification of Tyr transport system and the acetic acid biosynthesis pathway, expression of phosphoketolase (*fpk*) gene from *Bifidobacterium adolescens* with endogenous phosphotransacetylase (*pta*) gene and engineering of cofactor balance together with adaptive evolution to confer acid resistance in Tyr-producing strain of *E. coli*, in which the shikimate pathway and AAA biosynthesis pathway were modulated, enhanced Tyr production and Tyr production by the engineered strain reached 92.8 g L^–1^ in fed-batch cultivation using a jar fermenter (Ping et al. 2023).

In *C. glutamicum*, Kurpejović et al. (2023) engineered a Tyr-producing strain by modifying the shikimate pathway. In the modified strain, mutant *aroG* was expressed and the initiation codons for *pheA* and *trpE* were replaced with a minor initiation codon (TTG) to decrease their translation efficiency; the resulting strain produced 3.1 g L^–1^ Tyr. However, unlike Phe production, modification of the PTS to enhance the supply of PEP did not improve Tyr production.

**Fig. 3:**
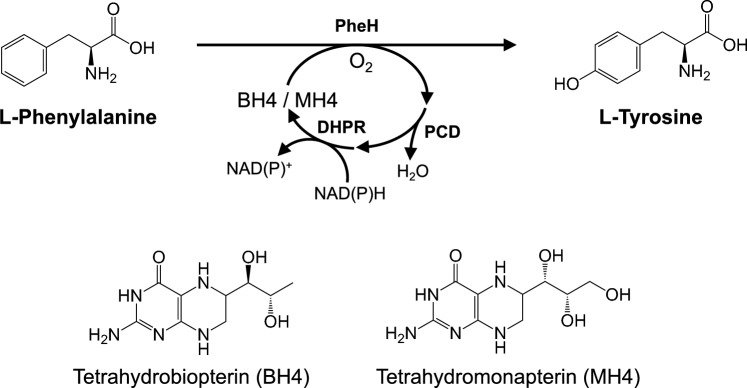
Tyrosine formation via hydroxylation of phenylalanine

The shikimate pathway is absent in humans and Phe and Trp are essential amino acids. Instead, Tyr is biosynthesized by the tetrahydrobiopterin (BH4)-dependent phenylalanine hydroxylase PheH, which catalyzes the formation of Tyr from Phe, molecular oxygen, and BH4 (Fitzpatrick 2023) (Fig. 3). Although some bacteria have homologs that use tetrahydromonapterin (MH4) as a cofactor (Pribat et al. 2010) (Fig. 3), these enzymes have never been used for fermentative production. The main challenge is the supply of tetrahydropterin, which is stoichiometrically consumed during the reaction in host cells. Satoh et al. (2012b) showed that this issue could be overcome by using the human BH4 regeneration system, which consists of pterin-4α-carbinolamine dehydratase (PCD) and dihydropteridine reductase (DHPR) (Fig. 3), when producing 3,4-dihydroxyphenylalanine (DOPA) with mouse tyrosine hydroxylase (TyrH), a homolog of PheH. Indeed, an engineered *E. coli* strain expressing these regeneration system-related genes and PheH from *Gulbenkiania* sp. SG4523, which was screened for high activity among eight enzymes from rat and bacteria, produced 4.63 g L^–1^ Tyr from 5 g L^–1^ of Phe in test tubes (Satoh et al. 2023). After further optimization and chromosomal engineering of *E. coli*, a strain was obtained that produced 5.19 g L^–1^ Tyr in test tube cultivation (Shen et al. 2024). Another group also reported production of Tyr (0.401 g L^–1^) from glucose by *E. coli* harboring PheH from *Xanthomonas campestris* and the MH4 regeneration system, including the PCD homolog PhhB from *Pseudomonas aeruginosa* and the dihydromonapterin reductase FolM from *E. coli*, in shake flasks (Huang et al. 2015), suggesting that this route is also available for Tyr production.

#### Tryptophan

Similar to Phe and Tyr production, Trp production in *E. coli* was engineered by improving metabolic flux of the shikimate pathway and Trp biosynthesis. Zhao et al. (2011) generated a Trp-producing strain of *E. coli* by enhancing flux of the shikimate and Trp biosynthesis pathways, avoiding Trp degradation, and blocking the competing Phe and Tyr biosynthesis pathways; the resulting strain produced about 13 g L^–1^ Trp in fed-batch cultivation. Gu et al. (2012) generated a Trp-producing strain of *E. coli* by expressing *tktA* encoding transketolase to improve the supply of E4P for enhancing shikimate pathway flux, and preventing Trp degradation; the resulting strain produced 1.3 g L^–1^ Trp in batch cultivation. Trp production in the strain was further enhanced by replacing the leader sequence and *trpEDCBA* operon promoter with a stronger promoter to about 1.7 and 10 g L^–1^ Trp in batch and fed-batch cultivation, respectively. Interestingly, Trp production by this strain was further improved by expressing polyhydroxybutyrate (PHB) biosynthesis genes from *Cupriavidus necator* (Gu et al. 2013). In the Trp production strain expressing heterologous PHB biosynthesis genes, expression of the *trpDCBA* genes were upregulated compared with that in the parental strain and this phenomenon may result in improved Trp production. However, the mechanism of upregulation of *trpDCBA* expression is not understood.

Liu et al. (2012) investigated the effect of deletion of *aroP* gene, which encodes AAA permease, and expression of *yddG* gene, which encodes an aromatic amino acid exporter, on Trp production in a Trp-producing *E. coli* strain. Wang et al. (2013) reported that deletion of *pta* and *mtr* encoding phosphotransacetylase and a high-affinity Trp transporter, respectively, combined with overexpression of *yddG*, reduced acetate production as a by-product and increased Trp production.

Li et al. (2020) reported the effects of optimizing the supply of precursor and cofactor on Trp production in *E. coli*. Trp biosynthesis requires L-glutamine, L-serine, and PRPP (Fig. 2). In this study, heterologous gene encoding glutamine synthetase and the endogenous *icd* and *gdhA* genes encoding isocitrate dehydrogenase and glutamate dehydrogenase, respectively, were expressed in an engineered Trp-producing strain to enhance the L-glutamine supply. Introduction of additional copies of the *prs* gene encoding phosphoribosylpyrophosphate synthase into the genome for improving PRPP supply and expression of mutant *serA* and *thrA* genes encoding 3‐phosphoglycerate dehydrogenase and bifunctional aspartokinase/homoserine dehydrogenase, respectively, with feedback resistance to L-serine for improving L-serine supply were additionally conducted; L-serine inhibits the activity of aspartokinase/homoserine dehydrogenase encoded by *thrA* and expression of mutant *thrA* gene is expected to maintain L-threonine biosynthesis even if L-serine supply is enhanced. To maintain redox balance, genes encoding transhydrogenases, which catalyze the interconversion of NADPH + NAD^+^ and NADP^+^  + NADH, were overexpressed in the engineered strain. Trp production in the final engineered strain reached 1.7 g L^–1^ in batch cultivation.

Guo et al. (2022b) metabolically engineered *E. coli* to improve and optimize the supply of precursors and modify the membrane transporters for Trp production. Trp biosynthesis was improved by removing the negative transcription factor TrpR, preventing the formation of competing by-products (Phe and Tyr), and overexpressing the *trpEDCBA* operon, in which *trpE* was replaced with a mutant encoding a feedback-resistant anthranilate synthase. To enhance the supply of PEP, the pathways for production of acetate, formate, lactate, and ethanol were disrupted. Moreover, to optimize the supply of substrates for DAHPS (i.e. PEP and E4P), recombinant strains of *E. coli* expressing *ppsA*, *tktA*, and mutant *aroG* under promoters with different strengths were constructed, and Trp production was evaluated. In addition, the expression of *serA*, *serB*, and *serC* was optimized by combinatorial screening of promoters to improve the L-serine supply, and the *yggG* gene, encoding the Trp exporter, was overexpressed. The resulting engineered *E. coli* cells produced 52.1 g L^–1^ Trp.

### Fermentative production of various aromatic amino acid derivatives

**Fig. 4:**
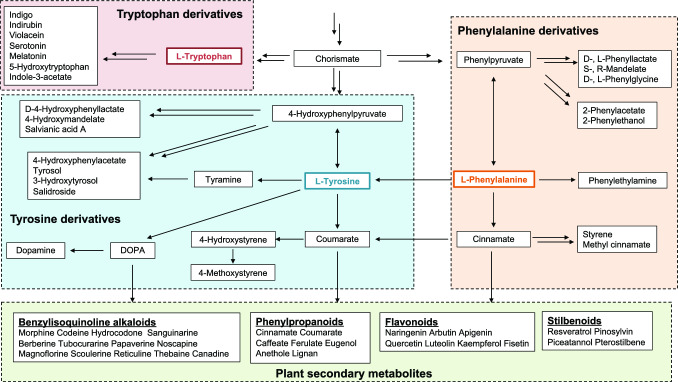
Synthetic pathways for various aromatic compounds from aromatic amino acids

AAAs are important starting compounds for the synthesis of various aromatic derivatives that are widely used in chemicals, food, polymers, and pharmaceuticals. Here, we briefly summarize fermentative production of these derivatives using *E. coli* and *C. glutamicum* (Fig. 4, Supplementary Figs. S2, S3, S4 and S5 and Supplementary Table S3).

#### Phenylalanine derivatives

In *E. coli*, phenylethylamine was produced by decarboxylation of Phe using aromatic amino acid decarboxylase from *Pseudomonas putida* (Koma et al. 2012a). Cinnamate, which is a starter unit of many secondary metabolites in plants such as phenylpropanoids, flavonoids, and stilbenoids, was produced at high levels (6.9 g L^–1^) by a Phe-overproducing *E. coli* strain overexpressing phenylalanine ammonia lyase gene in fed-batch cultivation (Bang et al. 2018). In *C. glutamicum*, de novo synthesis of cinnamate has not yet been reported, but successful bioconversion of Phe to cinnamate has been reported (Son et al. 2021). Cinnamate can be converted to styrene (the monomeric unit of the conventional plastic polystyrene) by cinnamate decarboxylase from *Saccharomyces cerevisiae* (McKenna and Nielsen 2011). In recent studies, styrene was produced at 3.1 and 5.3 g L^–1^ in two-phase batch cultivation (Noda et al. 2024a) and fed-batch cultivation combined with gas stripping technology (Lee et al. 2019), respectively. Cinnamate was also converted to methyl cinnamate, a fragrance ingredient with fruity balsamic odor, by overexpression of cinnamate carboxyl methyltransferase which transferred the methyl group from S-adenosylmethionine to cinnamate (Guo et al. 2022a).

Phe can be reversibly converted to phenylpyruvate via the aminotransferase reaction in vivo, and many aromatic derivatives can be synthesized from phenylpyruvate. In *S. cerevisiae*, phenylpyruvate can be converted by endogenous phenylpyruvate decarboxylase encoded by *ARO10* and aldehyde reductase to 2-phenylethanol, a compound with rose-like odor. Production of 2-phenylethanol from phenylpyruvate in *E. coli* was achieved by implementing the synthetic pathway from Phe, namely the Ehrlich pathway, which includes 2-keto acid decarboxylase and alcohol dehydrogenase. Koma et al. (2012b) constructed a 2-phenylethanol-overproducing strain of *E. coli* through expression of phenylpyruvate decarboxylase from *Azospirillum brasilense*, which is a counterpart of Aro10 from *S. cerevisiae*. High-level production of 2-phenylethanol requires deletion of *feaB*, which encodes phenylacetaldehyde dehydrogenase and is responsible for the accumulation of 2-phenylacetate. Guo et al. (2018) exploited the synthesis pathway including Aro10 in *E. coli* cells to produce 2-phenylethanol from glucose de novo. In a recent study, 2.5 g L^–1^ 2-phenylethanol was produced from glucose, with a yield of 0.16 g g-glucose^–1^, in batch cultivation using an *E. coli* strain harboring a mutant Aro10 from *S. cerevisiae* (Noda et al. 2024b). An engineered *C. glutamicum* strain produced 3.23 g L^–1^ 2-phenylethanol, with a yield of 0.05 g g-glucose^–1^ (Zhu et al. 2023).

Phenylpyruvate can be reduced to phenyllactate enantioselectivity, and D-phenyllactate-overproducing strains of *E. coli* were successfully generated by introducing D-lactate dehydrogenase from *Cupriavidus necator* or phenylpyruvate reductase from *Wickerhamia fluorescens* into a Phe-overproducing strain (Koma et al. 2012b; Fujita et al. 2013). Using L-lactate dehydrogenase instead of such dehydrogenase and reductase resulted in the production of L-phenyllactate. A recent study showed that 52.89 g L^–1^ phenyllactate was produced from glucose by an engineered *E. coli* strain in fed-batch cultivation (Wu et al. 2024).

Moreover, phenylpyruvate was converted to S-mandelate by 4-hydroxymandelate synthase from *Amycolatopsis orientalis* in *E. coli* and was further converted to R-mandelate by 4-hydroxymandelate oxidase from *Streptomyces coelicolor* and D-mandelate dehydrogenase from *Rhodotorula graminis* (Sun et al. 2011). By expanding S-madelate synthesis pathway, D- and L-phenylglycine were also synthesized de novo from glucose in *E. coli* through phenylglyoxylate formation (Müller et al. 2006; Liu et al. 2014).

#### Tyrosine derivatives

Similar to the Phe derivatives, Tyr derivatives, which possess a hydroxyl group at fourth position of the benzene ring, can be synthesized. Tyramine was synthesized from glucose in *E. coli* and *C. glutamicum* via Tyr following overexpression of the tyrosine decarboxylase from *Levilactobacillus brevis* (synonymous with *Lactobacillus brevis*) or *Enterococcus faecium* (Koma et al. 2012a; Yang et al. 2022; Poethe et al. 2024). In *E. coli*, 4-hydroxyphenylpyruvate, an intermediate metabolite in the Tyr biosynthesis pathway, was converted to 4-hydroxymandelate by 4-hydroxymandelate synthase from *Amycolatopsis orientalis* (Li et al. 2016). Also in *E. coli*, 4-hydroxyphenylpyruvate was further converted to D-4-hydroxyphenyllactate by exogenous D-lactate dehydrogenase (Koma et al. 2012b). D-4-Hydroxyphenyllactate was further converted to salvianic acid A, which is a bioactive ingredient extracted from *Salvia miltiorrhiza*, by the flavin-dependent 4-hydroxyphenylacetate 3-hydroxylase complex (HpaBC, see below), from glucose, and the production reached 7.1 g L^–1^ in fed-batch cultivation (Yao et al. 2013).

A coumarate-producing strain of *E. coli* was generated by overexpressing the Tyr ammonia lyase gene in Tyr-overproducing *E. coli*; this strain produced 3.1 g L^–1^ coumarate in fed-batch cultivation (Qiu et al. 2024). Coumarate was further converted to 4-hydroxystyrene, a potential polymer resource, by introducing exogenous *p*-hydroxycinnamate decarboxylase into *E. coli* (Qi et al. 2007). Although coumarate production by engineered *C. glutamicum* has also been reported, the production level was lower than that by engineered *E. coli* (Mutz et al. 2023). However, a *C. glutamicum* strain overexpressing the phenolate decarboxylase gene produced more 4-hydroxystyrene from coumarate than an engineered *E. coli* strain (Rodriguez et al. 2021). Recently, small amount of 4-methoxystyrene (4-vinylanisole), which is known as an aggregation pheromone of migratory locusts, was successfully synthesized in *E. coli* from glucose by methylation of the hydroxy group of 4-hydroxystyrene (Hu et al. 2024).

DOPA is an important neurotransmitter precursor that is used for treatment of Parkinson’s disease. DOPA is also a key compound in the production of benzylisoquinoline alkaloids, such as morphine, codeine, and thebaine (Fig. 4). Therefore, reconstruction of the DOPA-producing pathway in microorganisms for their fermentative production has attracted much attention. Tyrosinase, which is involved in the production of melanin pigments, is a candidate enzyme which can be used for fermentative DOPA production. This enzyme catalyzes a two-step oxidation reaction in which Tyr is converted to DOPA and then to dopaquinone, using O_2_ as an oxidant. Reduction of dopaquinone with a reducing agent L-ascorbate is required to produce DOPA by tyrosinase, because the latter reaction is faster than the former reaction (Ali et al. 2007). Nakagawa et al. (2011) and Kurpejović et al. (2021) succeeded in producing DOPA de novo in engineered *E. coli* and *C. glutamicum* strains, respectively. The HpaBC complex from *E. coli* W was also employed for DOPA production. HpaB catalyzes the *ortho*-hydroxylation reaction converting 4-hydroxyphenylacetate using O_2_ and FADH_2_, and HpaC oxidizes NADH to NAD^+^ to provide FADH_2_ as a cofactor for HpaB (Prieto et al. 1993; Lee and Xun 1998; Xun and Sandvik 2000). Because of its broad substrate specificity, HpaB has been used to produce various complex phenolic compounds. In a recent study, 25.53 g L^–1^ DOPA was produced from glucose using a Tyr-producing *E. coli* strain overexpressing an HpaBC mutant in fed-batch cultivation (Fordjour et al. 2019). Another production pathway involves BH4-dependent TyrH as described above (Satoh et al. 2012b, 2023). During the TyrH-catalyzed reaction, one oxygen atom in an oxygen molecule is used for *ortho*-hydroxylation of Tyr and the other oxygen atom oxidizes BH4, yielding DOPA as the product.

Tyrosol, an antioxidant found in olive oil, is produced from 4-hydroxyphenylpyruvate in *E. coli* via an artificial Ehrlich pathway (Chung et al. 2017). A 4-hydroxyphenylacetate-producing strain of *E. coli* was obtained by overexpressing *feaB* along with the exogenous phenylpyruvate decarboxylase gene (Koma et al. 2012b). In recent studies, 3.9 g L^–1^ tyrosol (Xu et al. 2020) and 28.57 g L^–1^ 4-hydroxyphenylacetate (Shen et al. 2021) were produced from glucose in fed-batch cultivation. Tyrosol was also synthesized through the tyramine route, which includes decarboxylation of Tyr, oxidative deamination of tyramine, and reduction of 4-hydroxyphenylacetaldehyde (Satoh et al. 2012a, 2023; Shen et al. 2024). 4-Hydroxyphenylacetaldehyde, a precursor of tyrosol, was also synthesized from Tyr by an aromatic acetaldehyde synthase from parsley in *E. coli* (Trantas et al. 2019; Yang et al. 2019). Oxidation of tyrosol by tyrosinase or HpaBC resulted in production of 3-hydroxytyrosol, which is a super antioxidant found in olives (Chung et al. 2017; Choo et al. 2018; Li et al. 2018; Deri-Zenaty et al. 2020). 3-Hydroxytyrosol is also synthesized via the DOPA route, which includes hydroxylation of Tyr, decarboxylation of DOPA, oxidative deamination of dopamine, and reduction of 3,4-dihydroxyphenylacetaldehyde (Satoh et al. 2012b, 2023). In recent studies, engineered *E. coli* strains have produced 3-hydroxytyrosol at 8.8 g L^–1^ from glucose (Koma et al. 2023) and 9.87 g L^–1^ from glycerol (Wang et al. 2023) in fed-batch cultivation. When a plant glycosyltransferase gene was introduced into a tyrosol-overproducing *E. coli* strain, a trace amount of salidroside (a tyrosol glucoside) was produced from glucose (Bai et al. 2014; Chung et al. 2017). Salidroside production was increased to 6.03 g L^–1^ in a co-culture system (Liu et al. 2018).

#### Tryptophan derivatives

Unlike Phe and Tyr, Trp has a unique chemical structure with an indole side chain and is used to obtain chemicals with unique characteristic properties. Indole production was confirmed in the native producer *E. coli*; Trp is converted to indole, pyruvate, and ammonia through a β-elimination reaction catalyzed by tryptophanase (TnaA) (Li and Young 2013). The low-level production in *E. coli* (0.7 g L^–1^) was considered to be due to the toxic effect of indole. Higher production was observed in indole-negative *C. glutamicum* expressing a heterologous *tnaA* gene, which avoids the toxicity through in situ product elimination in culture broth using a water-insoluble solvent (Mindt et al. 2022). This pathway can be extended to produce indigo and indirubin (Ameria et al. 2015). In addition, violacein, a natural violet pigment derived from Trp, was produced in both *E. coli* and *C. glutamicum* by overexpressing the biosynthetic gene operon *vioABCDE* of *Chromobacterium violaceum* (Sun et al. 2016; Yang et al. 2021).

In animals, Trp is a precursor of neurotransmitters such as serotonin (5-hydroxytryptamine) and melatonin. Their biosynthesis starts with hydroxylation of Trp to form 5-hydroxytryptophan (5-HTP) by BH4-dependent Trp hydroxylase (TrpH), a homolog of PheH and TyrH (Fitzpatrick 2023), in a reaction described above. Then, serotonin is synthesized from 5-HTP via decarboxylation by tryptophan decarboxylase. Serotonin is converted to melatonin from in a two-step reaction involving *N*-acetylation by serotonin *N*-acetyltransferase with acetyl-CoA and *O*-methylation by *S*-adenosyl-methionine-dependent *N*-acetylserotonin *O*-methyltransferase. To produce 5-HTP, Zhang et al. (2022) employed an engineered *E. coli* strain expressing TrpH enzyme with the human BH4 biosynthesis and regeneration pathways, which yielded 8.58 g L^–1^ 5-HTP in fed-batch fermentation on glucose as the carbon source. In addition, Lin et al. (2014) succeeded in producing 1.1 and 0.15 g L^–1^ 5-HTP from Trp and glucose, respectively, using *E. coli* harboring both bacterial PheH, which was engineered to accept Trp as a substrate, and an MH4 regeneration system in a shake flask. 5-HTP was also synthesized by overexpressing TrpB, which catalyzes the β-substitution reaction between indole and L-serine, in L-serine-producing *C. glutamicum* under 5-hydroxyindole feeding conditions (Ferrer et al. 2022). Serotonin (1.68 g L^–1^) and melatonin (2.0 g L^–1^) have been successfully synthesized in *E. coli* from Trp via 5-HTP (Luo et al. 2020; Shen et al. 2022). Furthermore, Luo et al. (2020) produced 1.0 g L^–1^ melatonin from glucose using *E. coli*.

Trp can also be used as a starter substrate for the biosynthesis of indole-3-acetate, the most important naturally occurring plant hormone with auxin activity. For its bioproduction, a biosynthetic pathway via indole-3-pyruvate (Supplementary Fig. S5) has been constructed in engineered *C. glutamicum*, which produced 7.3 g L^–1^ indole-3-acetate from glucose and Trp (total amount 10 g L^–1^) in 5 L bioreactor (Yu-mi et al. 2019). In addition, recombinant *E. coli* with the same pathway produced 3.0 g L^–1^ indole-3-acetate from 4.0 g L^–1^ Trp in flask culture (Romasi and Lee 2013). De novo indole-3-acetate production at 0.7 g L^–1^ from 20 g L^–1^ glucose by *E. coli* with enhanced Trp supply was also reported under flask culture conditions (Guo et al. 2019). Wu et al. (2021) successfully achieved higher indole-3-acetate production at 7.1 g L^–1^ from 10 g L^–1^ Trp by comparing the other indole-3-acetate-producing pathways via tryptamine and indole-3-acetamide (Supplementary Fig. S5). Furthermore, de novo indole-3-acetate biosynthesis using the indole-3-acetamide pathway was established by improving Trp supply and NAD(P)H availability.

## Conclusion and future perspectives

Recent studies have reported the production of AAAs and their derivatives using *E. coli* and *C. glutamicum* as well as other microorganisms. Production targets for AAA derivatives, including alkaloids from plants, have also recently expanded. Research efforts to date have mainly focused on building pathways to produce target compounds. For the industrial applications, it is essential to further improvements in titer, yield, and productivity. To achieve this, the toxic and inhibitory effects of target products and metabolic intermediates on host cells would need to be overcome through the application of cofactor balancing, transporter engineering, and adaptive laboratory evolution. In addition, computationally aided tools to predict and mitigate the effects would be required to facilitate rational engineering. *C. glutamicum*, which is highly tolerant to environmental stresses in nature, would be a preferable host.

For efficient production of AAA derivatives and expansion of production targets, the establishment of strategies for metabolic pathway design and enhancement of target productivity based on genome information and synthetic biology are essential. Particularly, highly established chromosome engineering technologies are desired as tools for synthetic biology. For the chromosome engineering of *E. coli* to delete genes for blocking of competing pathways and insert genes for flux control, the λRed recombination method with antibiotic resistance markers for selection of recombinant cells has typically been used. One disadvantage of this method is that multiple scars on the chromosomes are remained through repeated modifications. Recently, the scar-less chromosome engineering tools using the λRed recombination together with clustered regularly interspaced short palindromic repeats (CRISPR)/CRISPR-associated (Cas) systems is established and widely used for the development of aromatic derivative-producing strains for the last several years (Fordjour et al. 2019; Xu et al. 2020; Shen et al. 2021, 2022; Yang et al. 2022; Zhang et al. 2022; Wang et al. 2023; Noda et al. 2024b; Wu et al. 2024). These tools facilitate the generation of high-performance, genetically stable, and plasmid-free strains suitable for industrial applications. Although chromosome engineering tools for *C. glutamicum* based on CRISPR/Cas system have been developed (Chen et al. 2023; Kim et al. 2023; Lee et al. 2024), few example of chromosome engineering in *C. glutamicum* for producing aromatic amino acids and their derivatives was reported. Further development of chromosome engineering technologies in *C. glutamicum* are necessary for producing not only aromatic amino acids and their derivatives but also other compounds.

## Supplementary Information

Below is the link to the electronic supplementary material.

Supplementary file1 (PDF 784 KB)

## Data Availability

No datasets were generated or analysed during the current study.

## References

[CR1] Ali S, Shultz JL, Ikram Ul H (2007) High performance microbiological transformation of L-tyrosine to L-dopa by *Yarrowia lipolytica* NRRL-143. BMC Biotechnol 7:50. 10.1186/1472-6750-7-50 17705832 10.1186/1472-6750-7-50 PMC2042982

[CR2] Ameria SPL, Jung HS, Kim HS, Han SS, Kim HS, Lee JH (2015) Characterization of a flavin-containing monooxygenase from *Corynebacterium glutamicum* and its application to production of indigo and indirubin. Biotechnol Lett 37:1637–1644. 10.1007/s10529-015-1824-2 25851950 10.1007/s10529-015-1824-2

[CR3] Bai Y, Bi H, Zhuang Y, Liu C, Cai T, Liu X, Zhang X, Liu T, Ma Y (2014) Production of salidroside in metabolically engineered *Escherichia coli*. Sci Rep 4:6640. 10.1038/srep06640 25323006 10.1038/srep06640 PMC4200411

[CR4] Bailey JE (1991) Toward a science of metabolic engineering. Science 252:1668–1675. 10.1126/science.2047876 2047876 10.1126/science.2047876

[CR5] Bang HB, Lee K, Lee YJ, Jeong KJ (2018) High-level production of trans-cinnamic acid by fed-batch cultivation of *Escherichia coli*. Process Biochem 68:30–36. 10.1016/j.procbio.2018.01.026

[CR6] Brown KD (1968) Regulation of aromatic amino acid biosynthesis *Escherichia coli* K12. Genetics 60:31–48. 10.1093/genetics/60.1.31 4884590 10.1093/genetics/60.1.31 PMC1212032

[CR7] Brune I, Jochmann N, Brinkrolf K, Huser AT, Gerstmeir R, Eikmanns BJ, Kalinowski J, Puhler A, Tauch A (2007) The IclR-type transcriptional repressor LtbR regulates the expression of leucine and tryptophan biosynthesis genes in the amino acid producer *Corynebacterium glutamicum*. J Bacteriol 189:2720–2733. 10.1128/JB.01876-06 17259312 10.1128/JB.01876-06 PMC1855810

[CR8] Burschowsky D, Thorbjornsrud HV, Heim JB, Fahrig-Kamarauskaite JR, Wurth-Roderer K, Kast P, Krengel U (2018) Inter-enzyme allosteric regulation of chorismate mutase in *Corynebacterium glutamicum*: structural basis of feedback activation by Trp. Biochemistry 57:557–573. 10.1021/acs.biochem.7b01018 29178787 10.1021/acs.biochem.7b01018

[CR9] Chen R, Shi F, Xiang Y, Lai W, Ji G (2023) Establishment of CRISPR-Cpf1-assisted gene editing tool and engineering of 4-hydroxyisoleucine biosynthesis in *Corynebacterium glutamicum*. World J Microbiol Biotechnol 39:266. 10.1007/s11274-023-03705-1 37524856 10.1007/s11274-023-03705-1

[CR10] Choo HJ, Kim EJ, Kim SY, Lee Y, Kim BG, Ahn JH (2018) Microbial synthesis of hydroxytyrosol and hydroxysalidroside. Appl Biol Chem 61:295–301. 10.1007/s13765-018-0360-x

[CR11] Chung D, Kim SY, Ahn JH (2017) Production of three phenylethanoids, tyrosol, hydroxytyrosol, and salidroside, using plant genes expressing in *Escherichia coli*. Sci Rep 7:2578. 10.1038/s41598-017-02042-2 28566694 10.1038/s41598-017-02042-2 PMC5451403

[CR12] Deri-Zenaty B, Bachar S, Rebroš M, Fishman A (2020) A coupled enzymatic reaction of tyrosinase and glucose dehydrogenase for the production of hydroxytyrosol. Appl Microbiol Biotechnol 104:4945–4955. 10.1007/s00253-020-10594-z 32285177 10.1007/s00253-020-10594-z

[CR13] Ding D, Liu Y, Xu Y, Zheng P, Li H, Zhang D, Sun J (2016) Improving the production of L-phenylalanine by identifying key enzymes through multi-enzyme reaction system in vitro. Sci Rep 6:32208. 10.1038/srep32208 27558633 10.1038/srep32208 PMC4997321

[CR14] Dopheide TAA, Crewther P, Davidson BE (1972) Chorismate mutase-prephenate dehydratase from *Escherichia coli* K-12: II kinetic properties. J Biol Chem 247:4447–4452. 10.1016/S0021-9258(19)45005-9 4261395

[CR15] Fazel AM, Jensen RA (1979) Obligatory biosynthesis of L-tyrosine via the pretyrosine branchlet in coryneform bacteria. J Bacteriol 138:805–815. 10.1128/jb.138.3.805-815.1979 457594 10.1128/jb.138.3.805-815.1979 PMC218108

[CR16] Ferrer L, Elsaraf M, Mindt M, Wendisch VF (2022) L-Serine biosensor-controlled fermentative production of L-tryptophan derivatives by *Corynebacterium glutamicum*. Biology 11:744 35625472 10.3390/biology11050744 PMC9138238

[CR17] Fitzpatrick PF (2023) The aromatic amino acid hydroxylases: structures, catalysis, and regulation of phenylalanine hydroxylase, tyrosine hydroxylase, and tryptophan hydroxylase. Arch Biochem Biophys 735:109518. 10.1016/j.abb.2023.109518 36639008 10.1016/j.abb.2023.109518

[CR18] Fordjour E, Adipah FK, Zhou S, Du G, Zhou J (2019) Metabolic engineering of *Escherichia coli* BL21 (DE3) for de novo production of L-DOPA from D-glucose. Microb Cell Fact 18:74. 10.1186/s12934-019-1122-0 31023316 10.1186/s12934-019-1122-0 PMC6482505

[CR19] Fujita T, Nguyen HD, Ito T, Zhou S, Osada L, Tateyama S, Kaneko T, Takaya N (2013) Microbial monomers custom-synthesized to build true bio-derived aromatic polymers. Appl Microbiol Biotechnol 97:8887–8894. 10.1007/s00253-013-5078-4 23949992 10.1007/s00253-013-5078-4

[CR20] Gavini N, Pulakat L (1991) Role of translation of the *pheA* leader peptide coding region in attenuation regulation of the *Escherichia coli pheA* gene. J Bacteriol 173:4904–4907. 10.1128/jb.173.15.4904-4907.1991 1856183 10.1128/jb.173.15.4904-4907.1991 PMC208172

[CR21] Gu P, Yang F, Kang J, Wang Q, Qi Q (2012) One-step of tryptophan attenuator inactivation and promoter swapping to improve the production of L-tryptophan in *Escherichia coli*. Microb Cell Fact 11:30. 10.1186/1475-2859-11-30 22380540 10.1186/1475-2859-11-30 PMC3311589

[CR22] Gu P, Kang J, Yang F, Wang Q, Liang Q, Qi Q (2013) The improved L-tryptophan production in recombinant *Escherichia coli* by expressing the polyhydroxybutyrate synthesis pathway. Appl Microbiol Biotechnol 97:4121–4127. 10.1007/s00253-012-4665-0 23321909 10.1007/s00253-012-4665-0

[CR23] Guo D, Zhang L, Kong S, Liu Z, Li X, Pan H (2018) Metabolic engineering of *Escherichia coli* for production of 2-phenylethanol and 2-phenylethyl acetate from glucose. J Agric Food Chem 66:5886–5891. 10.1021/acs.jafc.8b01594 29808680 10.1021/acs.jafc.8b01594

[CR24] Guo D, Kong S, Chu X, Li X, Pan H (2019) De novo biosynthesis of indole-3-acetic acid in engineered *Escherichia coli*. J Agric Food Chem 67:8186–8190. 10.1021/acs.jafc.9b02048 31272146 10.1021/acs.jafc.9b02048

[CR25] Guo D, Wu S, Fu X, Pan H (2022a) *De novo* biosynthesis of methyl cinnamate in engineered *Escherichia coli*. J Agric Food Chem 70:7736–7741. 10.1021/acs.jafc.2c02638 35709502 10.1021/acs.jafc.2c02638

[CR26] Guo L, Ding S, Liu Y, Gao C, Hu G, Song W, Liu J, Chen X, Liu L (2022b) Enhancing tryptophan production by balancing precursors in *Escherichia coli*. Biotechnol Bioeng 119:983–993. 10.1002/bit.28019 34936092 10.1002/bit.28019

[CR27] Hagino H, Nakayama K (1973) L-Tyrosine production by analog-resistant prototrophic mutants of glutamic acid producing bacteria. Agric Biol Chem 37:2007–2011. 10.1271/bbb1961.37.2007

[CR28] Hagino H, Nakayama K (1974) L-Phenylalanine production by analog-resistant mutants of *Corynebacterium glutamicum*. Agric Biol Chem 38:157–161. 10.1080/00021369.1974.10861130

[CR29] Hagino H, Nakayama K (1975) L-Tryptophan production by analog-resistant mutants derived from a phenylalanine and tyrosine double auxotroph of *Corynebacterium glutamicum*. Agric Biol Chem 39:343–349. 10.1080/00021369.1975.10861621

[CR30] Hu C, Yang J, Guo W, Pan H, Guo D (2024) *De novo* biosynthesis of 4-vinylanisole in engineered *Escherichia coli*. J Agric Food Chem 72:4334–4338. 10.1021/acs.jafc.3c09297 38354400 10.1021/acs.jafc.3c09297

[CR31] Huang J, Lin Y, Yuan Q, Yan Y (2015) Production of tyrosine through phenylalanine hydroxylation bypasses the intrinsic feedback inhibition in *Escherichia coli*. J Ind Microbiol Biotechnol 42:655–659. 10.1007/s10295-015-1591-z 25645094 10.1007/s10295-015-1591-z

[CR32] Hudson GS, Howlett GJ, Davidson BE (1983) The binding of tyrosine and NAD^+^ to chorismate mutase/prephenate dehydrogenase from *Escherichia coli* K12 and the effects of these ligands on the activity and self-association of the enzyme. Analysis in terms of a model. J Biol Chem 258:3114–3120. 10.1016/S0021-9258(18)32838-2 6338013

[CR33] Ito J, Crawford IP (1965) Regulation of the enzymes of the tryptophan pathway in *Escherichia coli*. Genetics 52:1303–1316. 10.1093/genetics/52.6.1303 5327408 10.1093/genetics/52.6.1303 PMC1210985

[CR34] Juminaga D, Baidoo EE, Redding-Johanson AM, Batth TS, Burd H, Mukhopadhyay A, Petzold CJ, Keasling JD (2012) Modular engineering of L-tyrosine production in *Escherichia coli*. Appl Environ Microbiol 78:89–98. 10.1128/AEM.06017-11 22020510 10.1128/AEM.06017-11 PMC3255607

[CR35] Kataoka N, Matsutani M, Matsushita K, Yakushi T (2023) Stepwise metabolic engineering of *Corynebacterium glutamicum* for the production of phenylalanine. J Gen Appl Microbiol 69:11–23. 10.2323/jgam.2022.08.002 35989300 10.2323/jgam.2022.08.002

[CR36] Kim HJ, Choi SS, Kim ES (2023) CRISPR-driven genome engineering for chorismate- and anthranilate-accumulating *Corynebacterium* Cell Factories. J Microbiol Biotechnol 33:1370–1375. 10.4014/jmb.2305.05031 37463859 10.4014/jmb.2305.05031 PMC10619553

[CR37] Kitade Y, Hashimoto R, Suda M, Hiraga K, Inui M (2018) Production of 4-hydroxybenzoic acid by an aerobic growth-arrested bioprocess using metabolically engineered *Corynebacterium glutamicum*. Appl Environ Microbiol. 10.1128/AEM.02587-17 29305513 10.1128/AEM.02587-17 PMC5835730

[CR38] Koma D, Yamanaka H, Moriyoshi K, Ohmoto T, Sakai K (2012a) A convenient method for multiple insertions of desired genes into target loci on the *Escherichia coli* chromosome. Appl Microbiol Biotechnol 93:815–829. 10.1007/s00253-011-3735-z 22127754 10.1007/s00253-011-3735-z

[CR39] Koma D, Yamanaka H, Moriyoshi K, Ohmoto T, Sakai K (2012b) Production of aromatic compounds by metabolically engineered *Escherichia coli* with an expanded shikimate pathway. Appl Environ Microbiol 78:6203–6216. 10.1128/aem.01148-12 22752168 10.1128/AEM.01148-12 PMC3416637

[CR40] Koma D, Fujisawa M, Ohashi H, Yamanaka H, Moriyoshi K, Nagamori E, Ohmoto T (2023) Production of 3-hydroxytyrosol from glucose by chromosomally engineered *Escherichia coli* by fed-batch cultivation in a jar fermenter. J Agric Food Chem 71:9451–9459. 10.1021/acs.jafc.3c02517 37279371 10.1021/acs.jafc.3c02517

[CR41] Kubota T, Tanaka Y, Takemoto N, Watanabe A, Hiraga K, Inui M, Yukawa H (2014) Chorismate-dependent transcriptional regulation of quinate/shikimate utilization genes by LysR-type transcriptional regulator QsuR in *Corynebacterium glutamicum*: carbon flow control at metabolic branch point. Mol Microbiol 92:356–368. 10.1111/mmi.12560 24674055 10.1111/mmi.12560

[CR42] Kubota T, Watanabe A, Suda M, Kogure T, Hiraga K, Inui M (2016) Production of *para*-aminobenzoate by genetically engineered *Corynebacterium glutamicum* and non-biological formation of an *N*-glucosyl byproduct. Metab Eng 38:322–330. 10.1016/j.ymben.2016.07.010 27471069 10.1016/j.ymben.2016.07.010

[CR43] Kurpejović E, Wendisch VF, Sariyar Akbulut B (2021) Tyrosinase-based production of L-DOPA by *Corynebacterium glutamicum*. Appl Microbiol Biotechnol 105:9103–9111. 10.1007/s00253-021-11681-5 34762142 10.1007/s00253-021-11681-5

[CR44] Kurpejović E, Burgardt A, Bastem GM, Junker N, Wendisch VF, Sariyar Akbulut B (2023) Metabolic engineering of *Corynebacterium glutamicum* for L-tyrosine production from glucose and xylose. J Biotechnol 363:8–16. 10.1016/j.jbiotec.2022.12.005 36566842 10.1016/j.jbiotec.2022.12.005

[CR45] Lee JY, Xun L (1998) Novel biological process for L-DOPA production from L-tyrosine by p-hydroxyphenylacetate 3-hydroxylase. Biotechnol Lett 20:479–482. 10.1023/A:1005440229420

[CR46] Lee K, Bang HB, Lee YH, Jeong KJ (2019) Enhanced production of styrene by engineered *Escherichia coli* and in situ product recovery (ISPR) with an organic solvent. Microb Cell Fact 18:79. 10.1186/s12934-019-1129-6 31053078 10.1186/s12934-019-1129-6 PMC6498506

[CR47] Lee DS, Cho EJ, Nguyen DT, Song Y, Chang J, Bae HJ (2024) Succinic acid production from softwood with genome-edited *Corynebacterium glutamicum* using the CRISPR-Cpf1 system. Biotechnol J 19:e2300309. 10.1002/biot.202300309 38180273 10.1002/biot.202300309

[CR48] Li G, Young KD (2013) Indole production by the tryptophanase TnaA in *Escherichia coli* is determined by the amount of exogenous tryptophan. Microbiology 159:402–410. 10.1099/mic.0.064139-0 23397453 10.1099/mic.0.064139-0

[CR49] Li PP, Li DF, Liu D, Liu YM, Liu C, Liu SJ (2013) Interaction between DAHP synthase and chorismate mutase endows new regulation on DAHP synthase activity in *Corynebacterium glutamicum*. Appl Microbiol Biotechnol 97:10373–10380. 10.1007/s00253-013-4806-0 23467831 10.1007/s00253-013-4806-0

[CR50] Li FF, Zhao Y, Li BZ, Qiao JJ, Zhao GR (2016) Engineering *Escherichia coli* for production of 4-hydroxymandelic acid using glucose-xylose mixture. Microb Cell Fact 15:90. 10.1186/s12934-016-0489-4 27234226 10.1186/s12934-016-0489-4 PMC4884394

[CR51] Li X, Chen Z, Wu Y, Yan Y, Sun X, Yuan Q (2018) Establishing an artificial pathway for efficient biosynthesis of hydroxytyrosol. ACS Synth Biol 7:647–654. 10.1021/acssynbio.7b00385 29281883 10.1021/acssynbio.7b00385

[CR52] Li Z, Ding D, Wang H, Liu L, Fang H, Chen T, Zhang D (2020) Engineering *Escherichia coli* to improve tryptophan production via genetic manipulation of precursor and cofactor pathways. Synth Syst Biotechnol 5:200–205. 10.1016/j.synbio.2020.06.009 32671235 10.1016/j.synbio.2020.06.009 PMC7334480

[CR53] Lin Y, Sun X, Yuan Q, Yan Y (2014) Engineering bacterial phenylalanine 4-hydroxylase for microbial synthesis of human neurotransmitter precursor 5-hydroxytryptophan. ACS Synth Biol 3:497–505. 10.1021/sb5002505 24936877 10.1021/sb5002505

[CR54] Liu YJ, Li PP, Zhao KX, Wang BJ, Jiang CY, Drake HL, Liu SJ (2008) *Corynebacterium glutamicum* contains 3-deoxy-D-arabino-heptulosonate 7-phosphate synthases that display novel biochemical features. Appl Environ Microbiol 74:5497–5503. 10.1128/AEM.00262-08 18621870 10.1128/AEM.00262-08 PMC2546626

[CR55] Liu Q, Cheng Y, Xie X, Xu Q, Chen N (2012) Modification of tryptophan transport system and its impact on production of L-tryptophan in *Escherichia coli*. Bioresour Technol 114:549–554. 10.1016/j.biortech.2012.02.088 22456235 10.1016/j.biortech.2012.02.088

[CR56] Liu SP, Xiao MR, Zhang L, Xu J, Ding ZY, Gu ZH, Shi GY (2013) Production of L-phenylalanine from glucose by metabolic engineering of wild type *Escherichia coli* W3110. Process Biochem 48:413–419. 10.1016/j.procbio.2013.02.016

[CR57] Liu SP, Liu RX, El-Rotail AA, Ding ZY, Gu ZH, Zhang L, Shi GY (2014) Heterologous pathway for the production of L-phenylglycine from glucose by *E. coli*. J Biotechnol 186:91–97. 10.1016/j.jbiotec.2014.06.033 25011099 10.1016/j.jbiotec.2014.06.033

[CR58] Liu X, Li XB, Jiang J, Liu ZN, Qiao B, Li FF, Cheng JS, Sun X, Yuan YJ, Qiao J, Zhao GR (2018) Convergent engineering of syntrophic *Escherichia coli* coculture for efficient production of glycosides. Metab Eng 47:243–253. 10.1016/j.ymben.2018.03.016 29596994 10.1016/j.ymben.2018.03.016

[CR59] Luo H, Schneider K, Christensen U, Lei Y, Herrgard M, Palsson BØ (2020) Microbial synthesis of human-hormone melatonin at gram scales. ACS Synth Biol 9:1240–1245. 10.1021/acssynbio.0c00065 32501000 10.1021/acssynbio.0c00065

[CR60] McKenna R, Nielsen DR (2011) Styrene biosynthesis from glucose by engineered *E. coli*. Metab Eng 13:544–554. 10.1016/j.ymben.2011.06.005 21722749 10.1016/j.ymben.2011.06.005

[CR61] Mindt M, Beyraghdar Kashkooli A, Suarez-Diez M, Ferrer L, Jilg T, Bosch D, Martins dos Santos V, Wendisch VF, Cankar K (2022) Production of indole by *Corynebacterium glutamicum* microbial cell factories for flavor and fragrance applications. Microb Cell Fact 21:45. 10.1186/s12934-022-01771-y 35331232 10.1186/s12934-022-01771-y PMC8944080

[CR62] Muday GK, Johnson DI, Somerville RL, Herrmann KM (1991) The tyrosine repressor negatively regulates *aroH* expression in *Escherichia coli*. J Bacteriol 173:3930–3932. 10.1128/jb.173.12.3930-3932.1991 1675635 10.1128/jb.173.12.3930-3932.1991 PMC208031

[CR63] Müller U, van Assema F, Gunsior M, Orf S, Kremer S, Schipper D, Wagemans A, Townsend CA, Sonke T, Bovenberg R, Wubbolts M (2006) Metabolic engineering of the *E. coli* L-phenylalanine pathway for the production of D-phenylglycine (D-Phg). Metab Eng 8:196–208. 10.1016/j.ymben.2005.12.001 16466681 10.1016/j.ymben.2005.12.001

[CR64] Mutz M, Kösters D, Wynands B, Wierckx N, Marienhagen J (2023) Microbial synthesis of the plant natural product precursor p-coumaric acid with *Corynebacterium glutamicum*. Microb Cell Fact 22:209. 10.1186/s12934-023-02222-y 37833813 10.1186/s12934-023-02222-y PMC10576375

[CR65] Nakagawa A, Minami H, Kim JS, Koyanagi T, Katayama T, Sato F, Kumagai H (2011) A bacterial platform for fermentative production of plant alkaloids. Nat Commun 2:326. 10.1038/ncomms1327 21610729 10.1038/ncomms1327 PMC3112539

[CR66] Neshat A, Mentz A, Ruckert C, Kalinowski J (2014) Transcriptome sequencing revealed the transcriptional organization at ribosome-mediated attenuation sites in *Corynebacterium glutamicum* and identified a novel attenuator involved in aromatic amino acid biosynthesis. J Biotechnol 190:55–63. 10.1016/j.jbiotec.2014.05.033 24910972 10.1016/j.jbiotec.2014.05.033

[CR67] Noda S, Fujiwara R, Mori Y, Dainin M, Shirai T, Kondo A (2024a) Styrene production in genetically engineered *Escherichia coli* in a two-phase culture. Biotech 13:2. 10.3390/biotech13010002 38247732 10.3390/biotech13010002 PMC10801462

[CR68] Noda S, Mori Y, Ogawa Y, Fujiwara R, Dainin M, Shirai T, Kondo A (2024b) Metabolic and enzymatic engineering approach for the production of 2-phenylethanol in engineered *Escherichia coli*. Bioresour Technol 406:130927. 10.1016/j.biortech.2024.130927 38830477 10.1016/j.biortech.2024.130927

[CR69] Patel N, Pierson DL, Jensen RA (1977) Dual enzymatic routes to L-tyrosine and L-phenylalanine via pretyrosine in *Pseudomonas aeruginosa*. J Biol Chem 252:5839–5846. 10.1016/S0021-9258(17)40099-8 407230

[CR70] Ping J, Wang L, Qin Z, Zhou Z, Zhou J (2023) Synergetic engineering of *Escherichia coli* for efficient production of L-tyrosine. Synth Syst Biotechnol 8:724–731. 10.1016/j.synbio.2023.10.005 38033756 10.1016/j.synbio.2023.10.005 PMC10686809

[CR71] Poethe SS, Junker N, Meyer F, Wendisch VF (2024) Sustainable production of the drug precursor tyramine by engineered *Corynebacterium glutamicum*. Appl Microbiol Biotechnol 108:499. 10.1007/s00253-024-13319-8 39476177 10.1007/s00253-024-13319-8 PMC11525245

[CR72] Pribat A, Blaby IK, Lara-Nunez A, Gregory JF 3rd, de Crecy-Lagard V, Hanson AD (2010) FolX and FolM are essential for tetrahydromonapterin synthesis in *Escherichia coli* and *Pseudomonas aeruginosa*. J Bacteriol 192:475–482. 10.1128/JB.01198-09 19897652 10.1128/JB.01198-09 PMC2805310

[CR73] Prieto MA, Perez-Aranda A, Garcia JL (1993) Characterization of an *Escherichia coli* aromatic hydroxylase with a broad substrate range. J Bacteriol 175:2162–2167. 10.1128/jb.175.7.2162-2167.1993 8458860 10.1128/jb.175.7.2162-2167.1993 PMC204336

[CR74] Qi WW, Vannelli T, Breinig S, Ben-Bassat A, Gatenby AA, Haynie SL, Sariaslani FS (2007) Functional expression of prokaryotic and eukaryotic genes in *Escherichia coli* for conversion of glucose to *p*-hydroxystyrene. Metab Eng 9:268–276. 10.1016/j.ymben.2007.01.002 17451990 10.1016/j.ymben.2007.01.002

[CR75] Qiu C, Wang X, Zuo J, Li R, Gao C, Chen X, Liu J, Wei W, Wu J, Hu G, Song W, Xu N, Liu L (2024) Systems engineering *Escherichia coli* for efficient production *p*-coumaric acid from glucose. Biotechnol Bioeng 121:2147–2162. 10.1002/bit.28721 38666765 10.1002/bit.28721

[CR76] Rodriguez A, Meadows JA, Sun N, Simmons BA, Gladden JM (2021) Evaluation of bacterial hosts for conversion of lignin-derived *p*-coumaric acid to 4-vinylphenol. Microb Cell Fact 20:181. 10.1186/s12934-021-01670-8 34526022 10.1186/s12934-021-01670-8 PMC8442356

[CR77] Romasi EF, Lee J (2013) Development of indole-3-acetic acid-producing *Escherichia coli* by functional expression of IpdC, AspC, and Iad1. J Microbiol Biotechnol 23:1726–1736. 10.4014/jmb.1308.08082 24043123 10.4014/jmb.1308.08082

[CR78] Satoh Y, Tajima K, Munekata M, Keasling JD, Lee TS (2012a) Engineering of a tyrosol-producing pathway, utilizing simple sugar and the central metabolic tyrosine, in *Escherichia coli*. J Agric Food Chem 60:979–984. 10.1021/jf203256f 22225426 10.1021/jf203256f

[CR79] Satoh Y, Tajima K, Munekata M, Keasling JD, Lee TS (2012b) Engineering of L-tyrosine oxidation in *Escherichia coli* and microbial production of hydroxytyrosol. Metab Eng 14:603–610. 10.1016/j.ymben.2012.08.002 22948011 10.1016/j.ymben.2012.08.002

[CR80] Satoh Y, Fukui K, Koma D, Shen N, Lee TS (2023) Engineered *Escherichia coli* platforms for tyrosine-derivative production from phenylalanine using phenylalanine hydroxylase and tetrahydrobiopterin-regeneration system. Biotechnol Biofuels Bioprod 16:115. 10.1186/s13068-023-02365-5 37464414 10.1186/s13068-023-02365-5 PMC10354952

[CR81] Shen YP, Liao YL, Lu Q, He X, Yan ZB, Liu JZ (2021) ATP and NADPH engineering of *Escherichia coli* to improve the production of 4-hydroxyphenylacetic acid using CRISPRi. Biotechnol Biofuels 14:100. 10.1186/s13068-021-01954-6 33879249 10.1186/s13068-021-01954-6 PMC8056492

[CR82] Shen P, Gu S, Jin D, Su Y, Wu H, Li Q, Yang J, He W, Huang J, Qi F (2022) Engineering metabolic pathways for cofactor self-sufficiency and serotonin production in *Escherichia coli*. ACS Synth Biol 11:2889–2900. 10.1021/acssynbio.2c00298 35866382 10.1021/acssynbio.2c00298

[CR83] Shen N, Satoh Y, Koma D, Ohashi H, Ogasawara Y, Dairi T (2024) Optimization of tyrosol-producing pathway with tyrosine decarboxylase and tyramine oxidase in high-tyrosine-producing *Escherichia coli*. J Biosci Bioeng 137:115–123. 10.1016/j.jbiosc.2023.12.002 38135638 10.1016/j.jbiosc.2023.12.002

[CR84] Shiio I, Sugimoto S, Kawamura K (1984) Production of L-tryptophan by sulfonamideresistant mutants. Agric Biol Chem 48:2073–2080. 10.1271/bbb1961.48.2073

[CR85] Son J, Jang JH, Choi IH, Lim CG, Jeon EJ, Bae Bang H, Jeong KJ (2021) Production of trans-cinnamic acid by whole-cell bioconversion from L-phenylalanine in engineered *Corynebacterium glutamicum*. Microb Cell Fact 20:145. 10.1186/s12934-021-01631-1 34303376 10.1186/s12934-021-01631-1 PMC8310591

[CR86] Springer M, Mayaux JF, Fayat G, Plumbridge JA, Graffe M, Blanquet S, Grunberg-Manago M (1985) Attenuation control of the *Escherichia coli* phenylalanyl-tRNA synthetase operon. J Mol Biol 181:467–478. 10.1016/0022-2836(85)90420-6 3158742 10.1016/0022-2836(85)90420-6

[CR87] Stephanopoulos G, Vallino JJ (1991) Network rigidity and metabolic engineering in metabolite overproduction. Science 252:1675–1681. 10.1126/science.1904627 1904627 10.1126/science.1904627

[CR88] Sun Z, Ning Y, Liu L, Liu Y, Sun B, Jiang W, Yang C, Yang S (2011) Metabolic engineering of the L-phenylalanine pathway in *Escherichia coli* for the production of S- or R-mandelic acid. Microb Cell Fact 10:71. 10.1186/1475-2859-10-71 21910908 10.1186/1475-2859-10-71 PMC3182895

[CR89] Sun H, Zhao D, Xiong B, Zhang C, Bi C (2016) Engineering *Corynebacterium glutamicum* for violacein hyper production. Microb Cell Fact 15:148. 10.1186/s12934-016-0545-0 27557730 10.1186/s12934-016-0545-0 PMC4997675

[CR90] Tachikawa Y, Okuno M, Itoh T, Hirasawa T (2024) Metabolic engineering with adaptive laboratory evolution for phenylalanine production by *Corynebacterium glutamicum*. J Biosci Bioeng 137:344–353. 10.1016/j.jbiosc.2024.01.006 38365536 10.1016/j.jbiosc.2024.01.006

[CR91] Teramoto H, Inui M, Yukawa H (2009) Regulation of expression of genes involved in quinate and shikimate utilization in *Corynebacterium glutamicum*. Appl Environ Microbiol 75:3461–3468. 10.1128/AEM.00163-09 19376919 10.1128/AEM.00163-09 PMC2687277

[CR92] Trantas E, Navakoudis E, Pavlidis T, Nikou T, Halabalaki M, Skaltsounis L, Ververidis F (2019) Dual pathway for metabolic engineering of *Escherichia coli* to produce the highly valuable hydroxytyrosol. PLoS ONE 14:e0212243. 10.1371/journal.pone.0212243 31682615 10.1371/journal.pone.0212243 PMC6828502

[CR93] Wang J, Cheng LK, Wang J, Liu Q, Shen T, Chen N (2013) Genetic engineering of *Escherichia coli* to enhance production of L-tryptophan. Appl Microbiol Biotechnol 97:7587–7596. 10.1007/s00253-013-5026-3 23775271 10.1007/s00253-013-5026-3

[CR94] Wang H, Wang L, Chen J, Hu M, Fang F, Zhou J (2023) Promoting FADH_2_ regeneration of hydroxylation for high-level production of hydroxytyrosol from glycerol in *Escherichia coli*. J Agric Food Chem 71:16681–16690. 10.1021/acs.jafc.3c05477 37877749 10.1021/acs.jafc.3c05477

[CR95] Wang X, Qiu C, Chen C, Gao C, Wei W, Song W, Wu J, Liu L, Chen X (2024) Metabolic engineering of *Escherichia coli* for high-level production of L-phenylalanine. J Agric Food Chem 72:11029–11040. 10.1021/acs.jafc.4c01563 38699920 10.1021/acs.jafc.4c01563

[CR96] Wu J, Liu Y, Zhao S, Sun J, Jin Z, Zhang D (2019) Application of dynamic regulation to increase L-phenylalanine production in *Escherichia coli*. J Microbiol Biotechnol 29:923–932. 10.4014/jmb.1901.01058 31154747 10.4014/jmb.1901.01058

[CR97] Wu H, Yang J, Shen P, Li Q, Wu W, Jiang X, Qin L, Huang J, Cao X, Qi F (2021) High-level production of indole-3-acetic acid in the metabolically engineered *Escherichia coli*. J Agric Food Chem 69:1916–1924. 10.1021/acs.jafc.0c08141 33541074 10.1021/acs.jafc.0c08141

[CR98] Wu W, Chen M, Li C, Zhong J, Xie R, Pan Z, Lin J, Qi F (2024) Efficient production of phenyllactic acid in *Escherichia coli* via metabolic engineering and fermentation optimization strategies. Front Microbiol 15:1457628. 10.3389/fmicb.2024.1457628 39247693 10.3389/fmicb.2024.1457628 PMC11377314

[CR99] Xu W, Yang C, Xia Y, Zhang L, Liu C, Yang H, Shen W, Chen X (2020) High-level production of tyrosol with noninduced recombinant *Escherichia coli* by metabolic engineering. J Agric Food Chem 68:4616–4623. 10.1021/acs.jafc.9b07610 32208625 10.1021/acs.jafc.9b07610

[CR100] Xun L, Sandvik ER (2000) Characterization of 4-hydroxyphenylacetate 3-hydroxylase (HpaB) of *Escherichia coli* as a reduced flavin adenine dinucleotide-utilizing monooxygenase. Appl Environ Microbiol 66:481–486. 10.1128/AEM.66.2.481-486.2000 10653707 10.1128/aem.66.2.481-486.2000 PMC91852

[CR101] Yang H, Xue Y, Yang C, Shen W, Fan Y, Chen X (2019) Modular engineering of tyrosol production in *Escherichia coli*. J Agric Food Chem 67:3900–3908. 10.1021/acs.jafc.9b00227 30873833 10.1021/acs.jafc.9b00227

[CR102] Yang D, Park SY, Lee SY (2021) Production of rainbow colorants by metabolically engineered *Escherichia coli*. Adv Sci (Weinh) 8:e2100743. 10.1002/advs.202100743 34032018 10.1002/advs.202100743 PMC8261500

[CR103] Yang T, Wu P, Zhang Y, Cao M, Yuan J (2022) High-titre production of aromatic amines in metabolically engineered *Escherichia coli*. J Appl Microbiol 133:2931–2940. 10.1111/jam.15745 35938518 10.1111/jam.15745

[CR104] Yanofsky C (1981) Attenuation in the control of expression of bacterial operons. Nature 289:751–758. 10.1038/289751a0 7007895 10.1038/289751a0

[CR105] Yao YF, Wang CS, Qiao J, Zhao GR (2013) Metabolic engineering of *Escherichia coli* for production of salvianic acid A via an artificial biosynthetic pathway. Metab Eng 19:79–87. 10.1016/j.ymben.2013.06.001 23774671 10.1016/j.ymben.2013.06.001

[CR106] Yu-mi K, Mi-hyang K, Hee-sook K (2019) Production of indole-3-acetate in *Corynebacterium glutamicum* by heterologous expression of the indole-3-pyruvate pathway genes. Microbiol Biotechnol Lett 47:242–249. 10.4014/mbl.1901.01013

[CR107] Zhang C, Zhang J, Kang Z, Du G, Yu X, Wang T, Chen J (2013) Enhanced production of L-phenylalanine in *Corynebacterium glutamicum* due to the introduction of *Escherichia coli* wild-type gene *aroH*. J Ind Microbiol Biotechnol 40:643–651. 10.1007/s10295-013-1262-x 23526182 10.1007/s10295-013-1262-x

[CR108] Zhang C, Kang Z, Zhang J, Du G, Chen J, Yu X (2014) Construction and application of novel feedback-resistant 3-deoxy-D-arabino-heptulosonate-7-phosphate synthases by engineering the N-terminal domain for L-phenylalanine synthesis. FEMS Microbiol Lett 353:11–18. 10.1111/1574-6968.12397 24517515 10.1111/1574-6968.12397

[CR109] Zhang C, Zhang J, Kang Z, Du G, Chen J (2015) Rational engineering of multiple module pathways for the production of L-phenylalanine in *Corynebacterium glutamicum*. J Ind Microbiol Biotechnol 42:787–797. 10.1007/s10295-015-1593-x 25665502 10.1007/s10295-015-1593-x

[CR110] Zhang Z, Yu Z, Wang J, Yu Y, Li L, Sun P, Fan X, Xu Q (2022) Metabolic engineering of *Escherichia coli* for efficient production of L-5-hydroxytryptophan from glucose. Microb Cell Fact 21:198. 10.1186/s12934-022-01920-3 36153615 10.1186/s12934-022-01920-3 PMC9509612

[CR111] Zhao ZJ, Zou C, Zhu YX, Dai J, Chen S, Wu D, Wu J, Chen J (2011) Development of L-tryptophan production strains by defined genetic modification in *Escherichia coli*. J Ind Microbiol Biotechnol 38:1921–1929. 10.1007/s10295-011-0978-8 21541714 10.1007/s10295-011-0978-8

[CR112] Zhu N, Xia W, Wang G, Song Y, Gao X, Liang J, Wang Y (2023) Engineering *Corynebacterium glutamicum* for de novo production of 2-phenylethanol from lignocellulosic biomass hydrolysate. Biotechnol Biofuels Bioprod 16:75. 10.1186/s13068-023-02327-x 37143059 10.1186/s13068-023-02327-x PMC10158149

[CR113] Zurawski G, Brown K, Killingly D, Yanofsky C (1978) Nucleotide sequence of the leader region of the phenylalanine operon of *Escherichia coli*. Proc Natl Acad Sci USA 75:4271–4275. 10.1073/pnas.75.9.4271 360214 10.1073/pnas.75.9.4271 PMC336095

