# Supplementary material for: Production of aromatic amino acids and their derivatives by Escherichia coli and Corynebacterium glutamicum
Source: World J Microbiol Biotechnol. 2025 Feb 7;41(2):65. doi: 10.1007/s11274-025-04264-3 (PMC11802643; doi:10.1007/s11274-025-04264-3)
Supplement: Supplementary file 1 — Supplementary file1 (PDF 784 KB) [file 11274_2025_4264_MOESM1_ESM.pdf]

Supplementary materials

Production of aromatic amino acids and their derivatives by *Escherichia coli* and *Corynebacterium glutamicum*

Takashi Hirasawa,[1,*] Yasuharu Satoh,[2] Daisuke Koma[3]

[1]School of Life Science and Technology, Institute of Science Tokyo, 4259 Nagatsuta-cho, Midori-ku, Yokohama, Kanagawa 226-8501, Japan

[2]Faculty of Engineering, Hokkaido University, N13 & W8, Kita-ku, Sapporo, Hokkaido 060-8628, Japan

[3]Osaka Research Institute of Industrial Science and Technology, 1-6-50 Morinomiya, Joto-ku, Osaka 536-8553, Japan.

*Corresponding author

thirasawa@life.isct.ac.jp

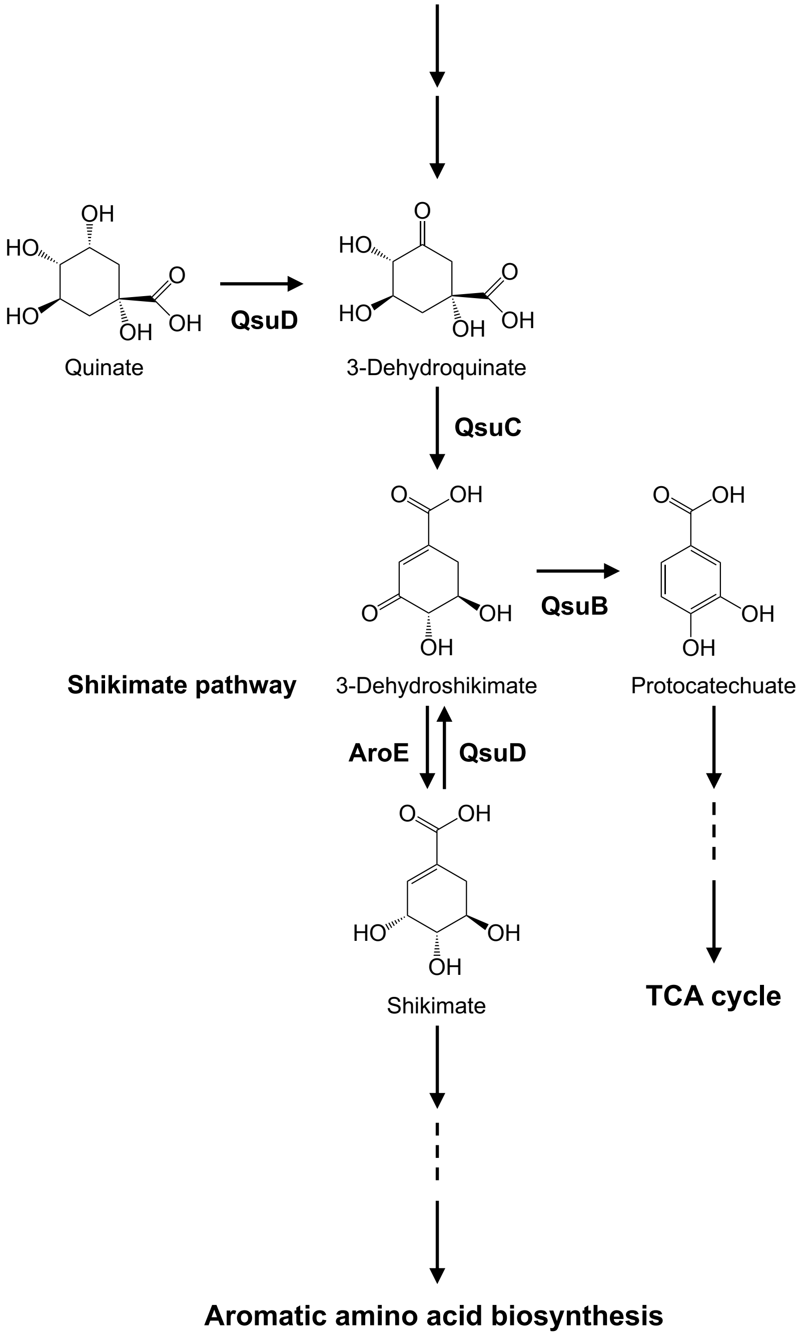

Supplementary Fig. S1 Metabolic pathway for assimilation of quinate and shikimate in *Corynebacterium glutamicum*.

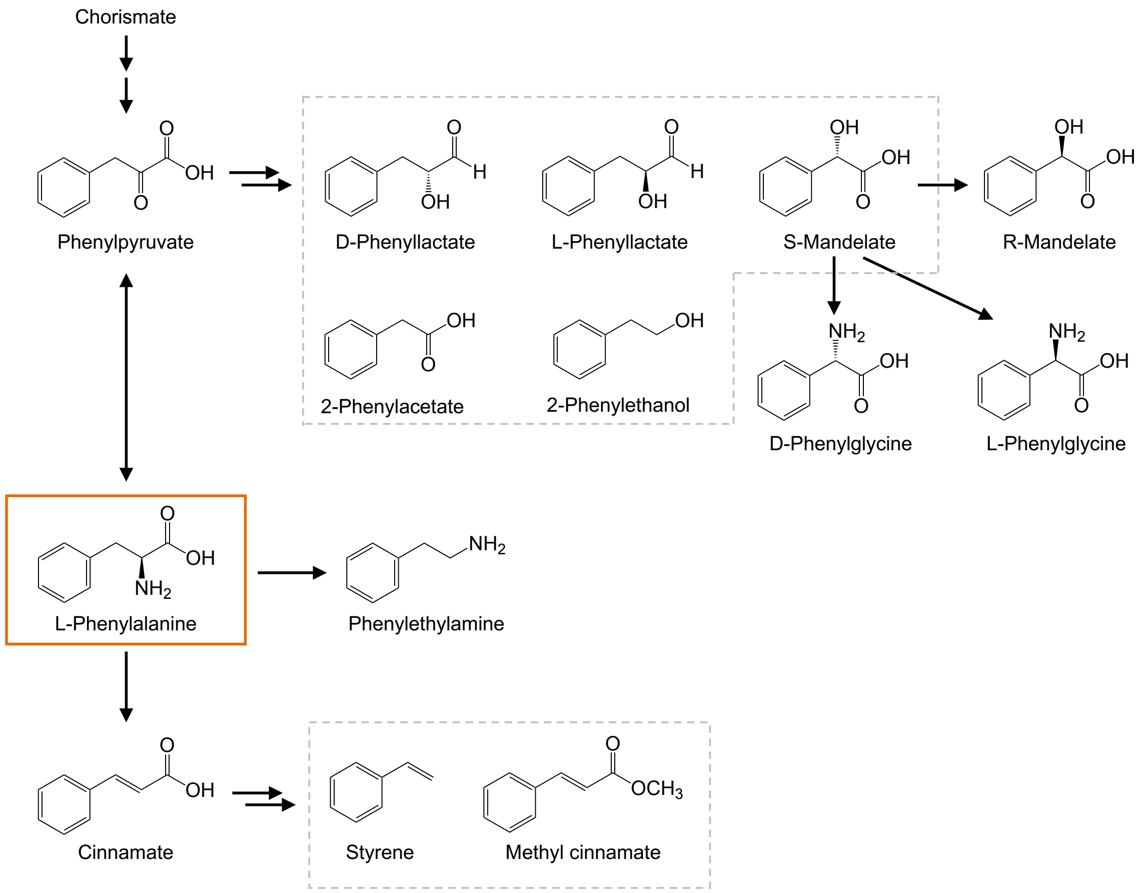

Supplementary Fig. S2 Phenylalanine derivatives introduced in this review.

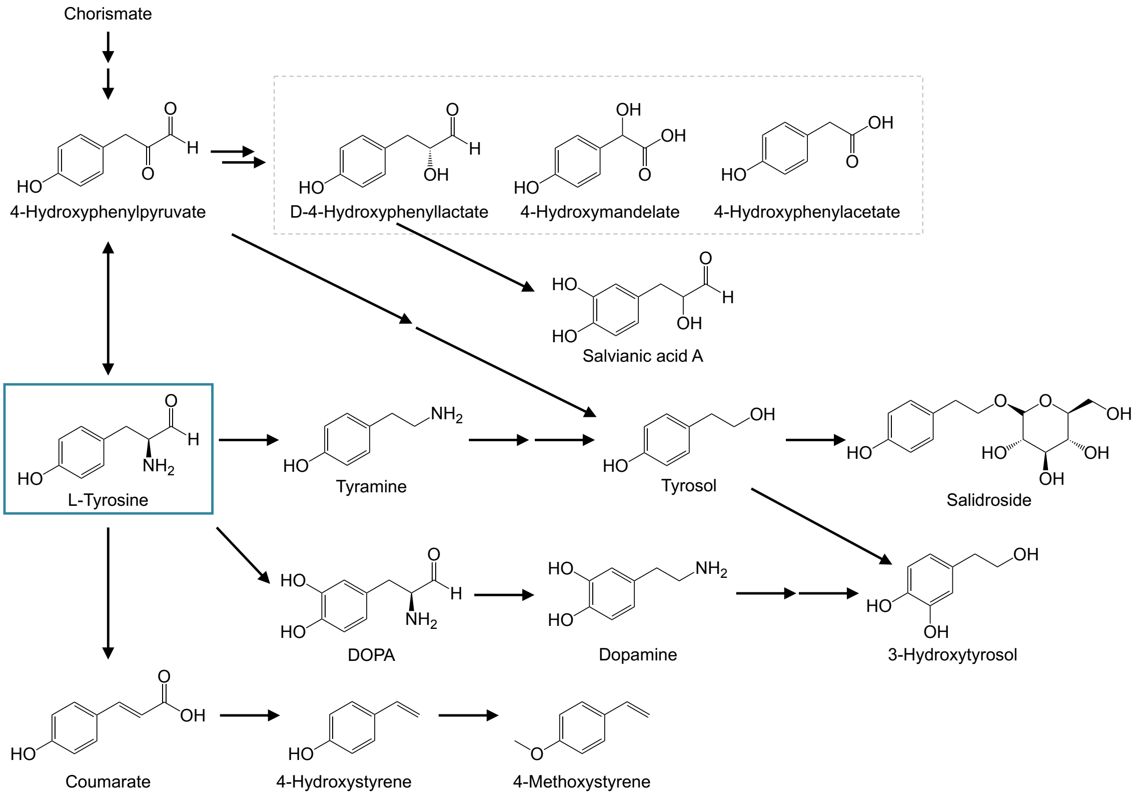

Supplementary Fig. S3 Tyrosine derivatives introduced in this review.

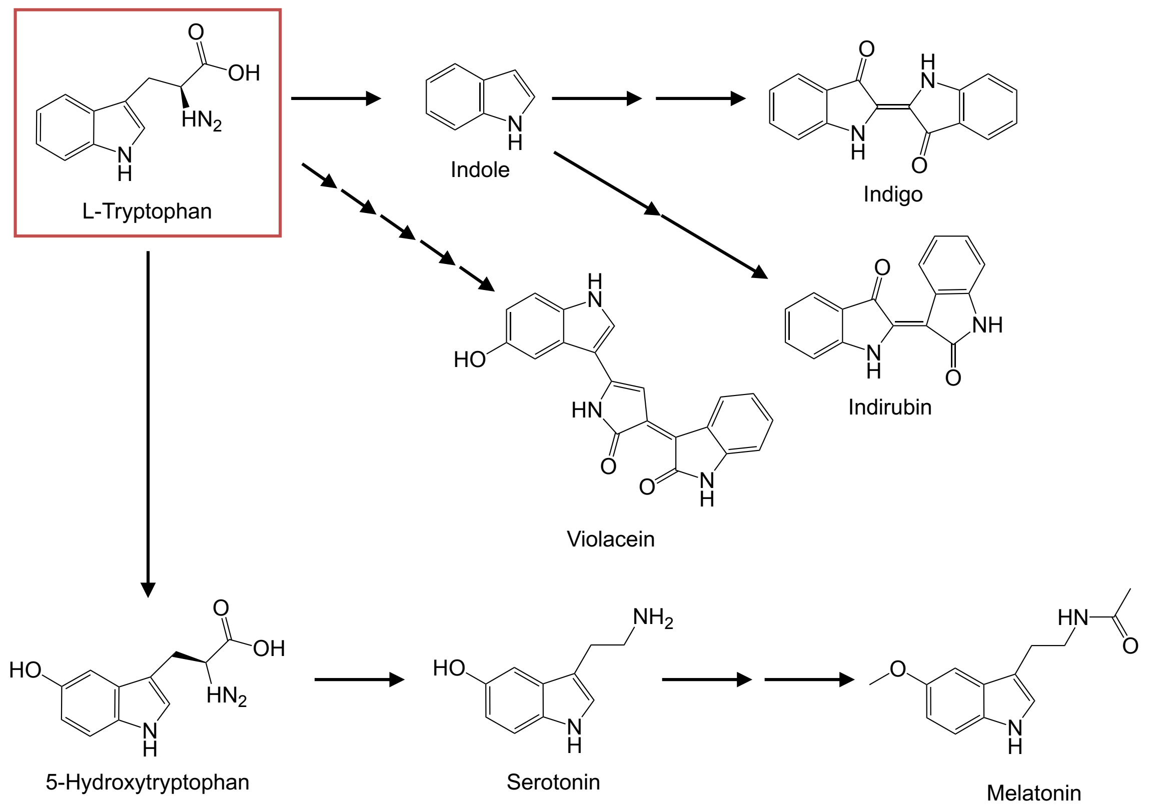

Supplementary Fig. S4 Tryptophan derivatives introduced in this review.

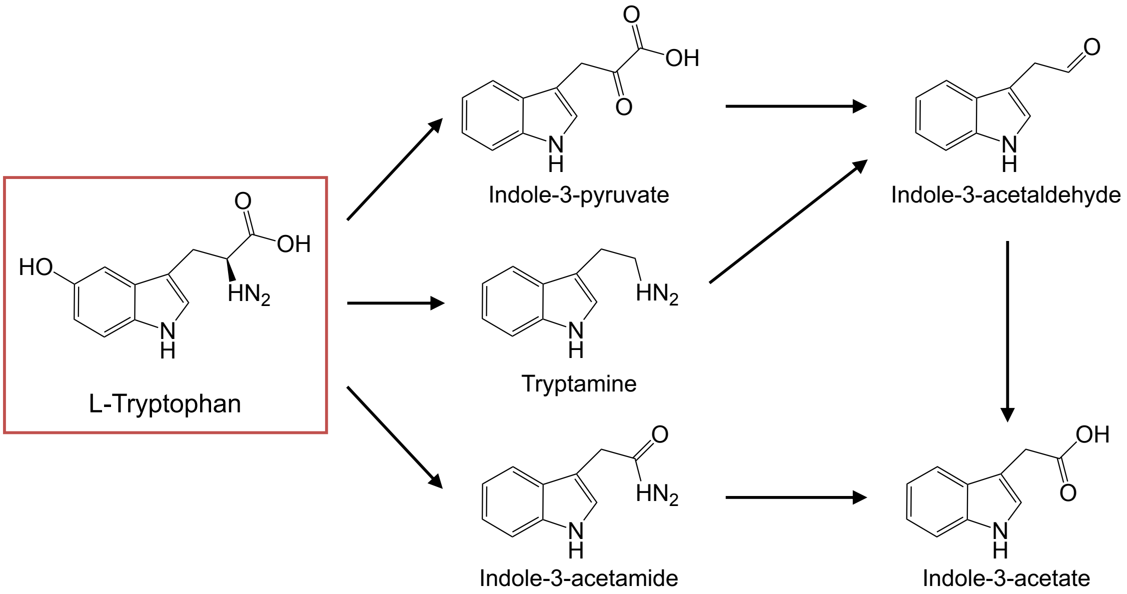

Supplementary Fig. S5 Reactions for producing indole-3-acetate from L-tryptophan in recombinant *E. coli* and *C. glutamicum*

Supplementary Table S1 List of enzymes, substrates, products and genes for aromatic amino acid biosynthesis pathways in *Escherichia coli* and *Corynebacterium glutamicum*.

Reaction No.	Enzyme	Substrate(s)	Product(s)	Gene(s)	
				E. coli	*C. glutamicum*
Chorismate biosynthesis					
1	3-Deoxy-D-arabinoheptulosonate-7-phosphate synthase	Phosphoenolpyruvate, erythrose-4-phosphate	3-Deoxy-D-arabinoheptulosonate-7-phosphate, phosphate	*aroG*, *aroF*, *aroH*	*aroF*, *aroG*
2	3-Dehydroquinate synthase	3-Deoxy-D-arabinoheptulosonate-7-phosphate	3-Dehydroquinate, phosphate	*aroB*	*aroB*
3	3-Dehydroquinate dehydratase	3-Dehydroquinate	3-Dehydroshikimate	*aroD*	*qsuC*
4	Shikimate dehydrogenase	3-Dehydroshikimate, $NAD(P)^+$	Shikimate, NAD(P)H	*aroE*, *ydiB*	*aroE*
5	Shikimate kinase	Shikimate, ATP	Shikimate-3-phosphate, ADP	*aroK*, *aroL*	*aroK*
6	5-Enolpyruvylshikimate-3-phosphate synthase	Shikimate-3-phosphate, phosphoenolpyruvate	5-Enolpyruvylshikimate-3-phosphate, phosphate	*aroA*	*aroA*
7	Chorismate synthase	5-Enolpyruvylshikimate-3-phosphate	Chorismate	*aroC*	*aroC*
Phe and Tyr biosynthesis from chorismate					
8	Chorismate mutase	Chorismate	Prephenate	*pheA*, *tyrA*	*csm*
9	Prephenate dehydratase	Prephenate	Phenylpyruvate, H_2O, CO_2	*pheA*	*pheA*
10	Prephenate dehydrogenase	Prephenate, $NAD(P)^+$	4-Hydroxyphenylpyruvate, NAD(P)H	*tyrA*	*tyrA*
11	Aminotransferase	Phenylpyruvate, amino donor (L-glutamate, L-alanine)	Phe, 2-oxoacid (2-oxoglutarate, pyruvate)	*tyrB*, *aspC*, *ilvE*	*pat*, *hisC*
		4-Hydroxyphenylpyruvate, amino donor (L-glutamate, L-alanine)	Tyr, 2-oxoacid (2-oxoglutarate, pyruvate)		
Trp biosynthesis from chorismate					
12	Anthranilate synthase	Chorismate, L-glutamine	Anthranilate, pyruvate, L-glutamate	*trpD*, *trpE*	*trpE*, *trpG*
13	Anthranilate phosphoribosyltransferase	Anthranilate, phosphoribosylpyrophosphate	Phosphoribosylanthranilate, pyrophosphate	*trpD*	*trpD*
14	Phosphoribosylanthranilate isomerase	Phosphoribosylanthranilate	Carboxyphenylaminodeoxy-D-ribulose-5-phosphate	*trpC*	*trpCF*
15	Indole-3-glycerol phosphate synthase	Carboxyphenylaminodeoxy-D-ribulose-5-phosphate	Indole-3-glycerol phosphate, H_2O, CO_2	*trpC*	*trpCF*, NCgl2010
16	Tryptophan synthase	Indole-3-glycerol phosphate, L-serine	Trp, glycelaldehyde-3-phosphate	*trpA*, *trpB*	*trpA*, *trpB*

Supplementary Table S2 Summary of studies on production of aromatic amino acids by *Escherichia coli* and *Corynebacterium glutamicum*

Production target	Host	Engineering strategy[a), b)]	Culture system	Carbon source	Productivity		Reference
					Titer (culture time)	Yield	
Phenylalanine	*E. coli*	Overexpression of the truncated *pheA* and *aroG*^{fbr} genes and increase in expression of the *ydiB* and *aroK*	Batch	Glucose	23.8 g L^{-1} (48 h)	0.153 g g-glucose^{-1}	Liu et al. 2013
	E. coli	Random mutagenesis, overexpression of the wild-type *aroF* and *aroA* genes and mutant *pheA*^{fbr} and *aroA*^{fbr} genes	Fed-batch	Glucose	62.47 g L^{-1} (48 h)	0.236 g g-glucose^{-1}	Ding et al. 2016
	E. coli	Replacement of the promoter for *aroK* with a mutant promoter for *tyrP* in the Phe-producing mutant obtained by random mutagenesis	Fed-batch	Glucose	61.3 g L^{-1} (48 h)	0.22 g g-glucose^{-1}	Wu et al. 2019
	E. coli	Expression of the heterologous genes *aroK1*, *aroL1*, and *pheA1* with the native *aroA*, *aroC*, and *tyrB* genes in the shikimate-producing strain and inserting *marA* into *tyrR* locus for overexpression	Fed-batch	Glucose	80.48 g L^{-1} (48 h)	0.27 g g-glucose^{-1}	Wang et al. 2024
	C. glutamicum	Overexpression of the wild-type *aroH* gene and the mutant *pheA*^{fbr} gene from *E. coli*	Batch	Glucose	4.64 g L^{-1} (not described)	Not described	Zhang et al. 2013
	C. glutamicum	Overexpression of truncated *aroF* gene from *C. glutamicum* and mutant *pheA*^{fbr} gene from *E. coli*	Batch	Glucose	4.29 g L^{-1} (72 h?)	Not described	Zhang et al. 2014
	C. glutamicum	Overexpression of mutant *aroF*^{fbr} and *pheA*^{fbr} with *aroA*, *aroE*, *aroL*, *tyrB*, *ppsA* and *tktA*, modification of phosphotransferase system, disruption of *aroP*, and blocking acetate and lactate formation	Fed-batch	Glucose	15.76 g L^{-1} (72 h?)	Not described	Zhang et al. 2015
	C. glutamicum	Overexpression of wild-type *aroH* and mutant *pheA*^{fbr} genes from *E. coli* on a plasmid and the *aroE* gene from the genome with disruption of *hdpA*, *qsuB*, *qsuD*, *tyrA*, and *ppc* genes	Batch	Glucose	50.7 mM (8.4 g L^{-1}) (33 h)	Not described	Kataoka et al. 2023
	C. glutamicum	Overexpression of mutant *aroG* and *pheA* genes from Phe analog-resistant *C. glutamicum* mutant obtained by adaptive laboratory evolution and disruption of *aroP*	Batch	Glucose	6.11 g L^{-1} (72 h)	Not described	Tachikawa et al. 2024
Tyrosine	*E. coli*	Expression of *aroE*, *aroD*, *aroB* (codon optimized), *aroG*^{fbr}, *ppsA*, *tktA*, *tyrB*, *tyrA*^{fbr},	Batch	Glucose	2.2 g L^{-1} (48 h)	Not described	Juminaga et al. 2012

		aroC, *aroA* and *aroL* cloned on medium copy number plasmids					
	E. coli	Overexpression of *aroG*^{fbr}, *ppsA*, *tktA*, *tyrA*^{fbr} and *yddG*, disruption of *tyrR*, *pheA*, *trpE* and *poxB*, expression of *fpk* from *Bifidobacterium adolescens* with endogenous *pta* gene, expression of *udhA* and *pntAB*, and adaptive laboratory evolution to confer acid resistance	Fed-batch	Glucose	92.8 g L^{-1} (62 h)	0.266 g g-glucose $^{-1}$	Ping et al. 2023
	E. coli	Expression of *pheH* from *Xanthomonas campestris* encoding phenylalanine hydroxylase, *phhB* from *Pseudomonas aeruginosa* encoding pterin-4α-carbinolamine dehydratase and *folM* from *E. coli* encoding dihydromonapterin reductase	Batch	Glucose	0.401 g L^{-1} (48 h?)	Not described	Huang et al. 2015
	C. glutamicum	Replacement of initiation codon for *pheA* and *trpE*, insertion of the mutant *aroG* gene from *E. coli* into *vdh* locus, and overexpression of *xylA* and *xylB* genes from *Xanthomonas campestris* and *C. glutamicum*, respectively, disruption of *ldh* gene	Batch	Glucose (10 g L^{-1}) + xylose (30 g L^{-1})	3.6 g L^{-1} (48 h)	90 mg g-glucose $^{-1}$	Kurpejović et al. 2023
Tryptophan	*E. coli*	Expression of mutant *aroF*^{fbr} and *trpE*^{fbr}*D* genes, disruption of *trpR* encoding a *trp* operon repressor, disruption of *tnaA* to reduce Trp degradation and disruption of *pheA* and *tyrA* to block Phe and Tyr formation	Fed-batch	Glucose	13.3 g L^{-1} (Not described)	0.10 g g-glucose $^{-1}$	Zhao et al. 2011
	E. coli	Disruption of *trpR* gene encoding *trp* operon repressor, expression of *aroG*^{fbr}, *trpE*^{fbr} and *tktA* genes cloned on a low-copy plasmid, disruption of *ptsG* to enhance phosphoenolpyruvate supply, disruption of *tnaA* gene to block Trp degradation, replacement of *trp* operon promoter and *trpL* with tac promoter	Batch<br>Fed-batch	Glucose<br>Glucose	1.7 g L^{-1} (36 h)<br>10.15 g L^{-1} (48 h)	Not described<br>Not described	Gu et al. 2012
	E. coli	Expression of polyhydroxybutyrate biosynthesis genes from *Cupriavidus necator* (formerly *Ralstonia eutropha*) in the Trp-producing strain constructed in the study by Gu et al. (2012)	Batch<br>Batch	Glucose<br>Glucose (16 g L^{-1}) + xylose (4 g L^{-1})	1.93 g L^{-1} (60 h)<br>2.24 g L^{-1} (60 h)	Not described<br>Not described	Gu et al. 2013
			Fed-batch	Glucose + xylose	14.4 g L^{-1} (72 h)	Not described	

E. coli	Overexpression of *yddG* encoding an amino acid exporter and disruption of *tnaA* in the Trp-producing strain of *E. coli* where *trpEDCBA* is overexpressed	Fed-batch	Glucose	36.3 g L^{-1} (36 h)	Not described	Liu et al. 2012
E. coli	Disruption of *pta* and *mtr* and overexpression of *yddG* in the Trp-producing strain where *aroG*^{fbr} and *trpE*^{fbr}*DBCA*, *serA*, *tktA* and *ppsA* on plasmids were expressed, *trpR* and *tnaA* were disrupted	Fed-batch	Glucose	48.68 g L^{-1} (38 h?)	Not described	Wang et al. 2013
E. coli	Expression of *aroG*^{fbr} and *trpE*^{fbr}*DCBA* genes on the plasmid, replacement of native *glnA* with *glnA* homolog from *Bacillus subtilis*, overexpression of *icd* and *gdhA* genes by promoter insertion, introduction of additional copy of *prs* into the genome, introduction of mutations into *serA* and *thrA* genes, overexpression of *sthA* and *pntAB* genes encoding transhydrogenases	Batch	Glucose	1.710 g L^{-1} (48 h)	Not described	Li et al. 2020
E. coli	Disruption of *trpR*, *tnaA*, *pheA* and *tyrA* genes, overexpression of *trpE*^{fbr}*DCBA* genes, blocking acetate, formate, lactate, and ethanol formation, expression of *ppsA*, *tktA*, and mutant *aroG* genes with different promoters on a single plasmid, expression of *serA*, *serB*, and *serC* with different promoters on a single plasmid, and disruption of *yggG* gene	Fed-batch	Glucose	52.1 g L^{-1} (36 h?)	0.171 g g-glucose $^{-1}$	Guo et al. 2022

Advanced studies reported after 2010 are summarized.

[a] fbr represents the gene encoding feedback-resistant enzyme.

[b] The respective homologous genes of *E. coli* and *C. glutamicum* are as follows: *aroA*, 5-enolpyruvylshikimate-3-phosphate synthase; *aroB*,3-dehydroquinate synthase; *aroC*, chorismate synthase; *aroD*, 3-dehydroquinate dehydratase; *aroE*, shikimate dehydrogenase; *aroF*, 3-deoxy-D-arabinoheptulosonate-7-phosphate synthase;, *aroG*, 3-deoxy-D-arabinoheptulosonate-7-phosphate synthase; *aroK*, shikimate kinase; *aroL*, shikimate kinase; *gdhA*, glutamate dehydrogenase; *glnA*, glutamine synthase; *icd*, isocitrate dehydrogenase; *ldh*, lactate dehydrogenase; *pheA*, chorismate mutase/prephenate dehydratase; *pntAB*, membrane transhydrogenase; *ppsA*, phosphoenolpyruvate synthetase; *prs*, phosphoribosylpyrophosphate synthase; *ptsG*, glucose-specific PTS enzyme IIBC component; *serA*, D-3-phosphoglycerate dehydrogenase, *serB*, phosphoserine phosphatase; *serC*, phosphoserine aminotransferase; *sthA*, soluble transhydrogenase; *thrA*, bifunctional aspartokinase/homoserine dehydrogenase; *tktA*, transketolase; *tnaA*, tryptophanase; *trpAB*, tryptophan synthase; *trpC*, anthranilate synthase; *trpDE*, anthranilate synthase; *trpR*; Trp operon repressor, *tyrA*; chorismate mutase/prephenate dehydrogenase, *tyrB*; aromatic amino acid aminotransferase, *tyrP*, Tyr-specific transporter; *tyrR*; DNA-binding transcriptional dual regulator, *yddG*; aromatic amino acid exporter, *ydiB*; shikimate dehydrogenase; *yggG*, Trp exporter.

Supplementary Table S3 Summary of studies on production of aromatic amino acid derivatives by *Escherichia coli* and *Corynebacterium glutamicum*

Production target	Host	Engineering strategy[a), b)]	Culture system	Carbon source	Production level		Reference
					Titer (culture time)	Yield	
Phe derivative							
D-Phenyllactate	*E. coli*	Overexpression of *aroG*^{fbr}, *pheA*^{fbr}, *tktA*, *pckA*, *glk* (glucose kinase, *Zymomonas mobilis*), *glf* (glucose permease, *Zymomonas mobilis*), *ppr* (phenylpyruvate reductase, *Lactobacillus* sp.) along with deletions of *tyrB*, *trpE*, *ptsH-ptsI-crr*.	Fed-batch	Glucose	52.89 g L^{-1} (48 h)	0.225 g g-glucose^{-1}	Wu et al. 2024
2-Phenylacetate	*E. coli*	Overexpression of *aroF*^{fbr}, *tyrA*^{fbr}, *feaB*, and *ipdC* (indole-pyruvate decarboxylase, *Azospirillum brasilense*) along with deletions of *tyrR*, *yahK*, and *pheA*.	Batch	Glucose	8.8 mM (48 h)	15.8% mol mol-glucose^{-1}	Koma et al. 2012b
2-Phenylethanol	*E. coli*	Overexpression of *glk*, *galP*, *yahK*, and *ARO10*^{I544W} (phenylpyruvate decarboxylase, *Saccharomyces cerevisiae*) along with deletions of *pykF*, *pykA*, *ptsHI* in Phe-overproducing strain ATCC31882.	Batch	Glucose	2.5 g L^{-1} (72 h)	0.16 g g-glucose^{-1}	Noda et al. 2024b
	C. glutamicum	Overexpression of *ARO10* (phenylpyruvate decarboxylase, *Saccharomyces cerevisiae*), *yahK*, *aroG*^{S180F} (*E. coli*), *pheA*^{fbr} (*E. coli*), *aroA*, *ppsA* (*E. coli*), *tktA* (*E. coli*) in 2-phenylethanol tolerance mutant of Phe overproducing strain ATCC21420. In addition, overexpression of *xylE* (pentose transporter, *E. coli*) and *xylAB* (xylose isomerase and xylulokinase, *Xanthomonas campestris*) for xylose or corn stalk hydrolysate as a substrate.	Batch<br>Batch<br>Batch	Glucose<br>Xylose<br>Corn stalk hydrolysate	3.23 g L^{-1} (60 h)<br>3.55 g L^{-1} (60 h)<br>3.28 g L^{-1} from corn stalk hydrolysate (<50 h)	0.05 g g-glucose^{-1}<br>0.06 g g-xylose^{-1}<br>Not described	Zhu et al. 2023
S-Mandelate	*E. coli*	Overexpression of *aroF*^{P148L}, *pheA*^{G309C}, *lacI*^{q}, and *hmaS* (4-hydroxymandelate synthase, *Amycolatopsis orientalis*) along with deletions of *tyrB*, *aspC*, *tyrA*, and *trpE*.	Batch	Glucose	0.74 g L^{-1} (24 h)<br>1.02 g L^{-1} (84 h)	6.5% g g-glucose $^{-1}$	Sun et al. 2011
R-Mandelate	*E. coli*	Overexpression of *hmo* (hydroxymandelate oxidase,	Batch	Glucose	0.68 g L^{-1} (24 h)<br>0.88 g L^{-1} (84 h)	7.8% g g-glucose^{-1}	Sun et al. 2011

		Streptomyces coelicolor) and *dmd* (D-mandelate dehydrogenase, *Rhodotorula graminis*) in S-mandelate producing strain (above).					
D-Phenylglycine	*E. coli*	Expression of *aroF*^{fbr}, overexpression of synthetic operon encoding *hmaS* (hydroxymandelate synthase, *Amycolatopsis orientalis*)-*hmo* (Hydroxymandelate oxidase, *Streptomyces coelicolor*)-*hpgAT* (phenylglycine aminotransferase, *Pseudomonas putida*)-*pheA*^{fbr} and deletions of *pheA*, *tyrA*, and *tyrR*.	Batch	Glucose	36 mg L^{-1} (24 h)	Not described	Müller et al. 2006
L-Phenylglycine	*E. coli*	Overexpression of *aroG15*, *pheA*^{fbr}, *aroK*, *ydiB*, *hmaS* (L-4-hydroxymandelate synthase), *hmo* (L-4-hydroxymandelate oxidase), and *hpgT* (L-4-hydroxyphenylglycine transaminase) along with deletions of *tyrA*, *crr*, *tyrB*, and *aspC*. The genes *hmaS*, *hmo*, and *hpgT* were derived from *Streptomyces coelicolor*.	Batch	Glucose	Not described	51.6 mg g-DCW^{-1}	Liu et al. 2014
Phenylethylamine	*E. coli*	Overexpression of *aroF*^{fbr}, *pheA*^{fbr}, and aromatic amino acid decarboxylase (*Pseudomonas putida*).	Batch	Glucose	4.8 mM (48 h)	Not described	Koma et al. 2012a
Cinnamate	*E. coli*	Overexpression of *glk*, *galP*, *SmPAL* (Phe ammonia-lyase, *Streptomyces maritimus*), *aroG*$^{D146N/A202T}$, *ydiB*, *aroK*, *pheA*$^{\Delta 301\text{-}386/E159A/E232A}$ along with deletions of *crr*, *tyrR*, *trpE*, *tyrA*, and *pykA*.	Fed-batch	Glucose	6.9 g L^{-1} (86 h)	0.028 g g-glucose^{-1}	Bang et al. 2018
Methyl cinnamate	*E. coli*	Overexpression of *aroG*^{fbr}, *tyrA*^{fbr}, *tktA*, *ppsA*, *metA*^{fbr}, *cysE*^{fbr}, *ccmt1* (cinnamate carboxyl methyltransferase 1, *Ocimum basilicum*), and Phe ammonia-lyase PA2 (laboratory stock).	Batch, two-phase culture system with dodecane	Glucose	458 mg L^{-1} (36 h)	Not described	Guo et al. 2022
Styrene	*E. coli*	Overexpression of *glk*, *galP*, *AtPAL2* (Phe ammonia-lyase, *Arabidopsis thaliana*) and *FDC1* (ferulate decarboxylase, *Saccharomyces cerevisiae*) along with deletions of *pykF*, *pykA*, *ptsHI* in Phe-overproducing strain ATCC31882.	Batch, biphasic culture	Glucose	3.1 g L^{-1} (96 h)	26.7% mol mol-glucose^{-1}	Noda et al. 2024a
	E. coli	Overexpression of *SmPAL* (Phe ammonia-lyase, *Streptomyces*	Fed-batch, in site product	Glucose	5.3 g L^{-1} (60 h)	Not described	Lee et al. 2019

		maritimus) and *ScFDC* (ferulic acid decarboxylase, *Saccharomyces cerevisiae*) along with deletions of *crr*, *tyrR*, *trpE*, *tyrA*, and *pykA*.	recovery using dodecane along with gas stripping system				
Tyr derivative							
4-Hydroxyphenyllactate	*E. coli*	Overexpression of *aroF*^{fbr}, *tyrA*^{fbr}, and *ldhA* (lactate dehydrogenase, *Cupriavidus necator*) along with deletion of *tyrR*.	Batch	Glucose	8.1 mM (48 h)	14.6% mol mol-glucose^{-1}	Koma et al. 2012b
4-Hydroxyphenylacetate	*E. coli*	Dynamic regulation using quorum-sensing-expression system for *ARO10* from *Saccharomyces cerevisiae* encoding phenylpyruvate decarboxylase and -repression system for *pabA* in the laboratory-stocked 4-hydroxyphenylacetate-overproducing and -tolerance strain generated by multiplex automated genome engineering and PCR-based random mutagenesis.	Fed-batch	Glucose	28.57 g L^{-1} (72 h)	27.64% mol mol-glucose^{-1}	Shen et al. 2021
Tyrosol	*E. coli*	Overexpression of *ARO10* from *Saccharomyces cerevisiae* along with deletions of *feaB*, *pheA*, *tyrB*, and *tyrR*.	Fed-batch	Glucose	3.9 g L^{-1} (48 h)	Not described	Xu et al. 2020
3-Hydroxytyrosol	*E. coli*	Chromosomal overexpression of *aroALC*, *aroEDB*, *aroG*^{fbr}, *tyrA*^{fbr}, *ppsA*, *tktA*, *hpaBC*, *yahK*, *lacI*^{q}, *ipdC* (indol-pyruvate decarboxylase, *Azospirillum brasilense*) along with deletion of *tyrR*, *adhE*, *ldhA*, *feaB*, *pykA*, *pykF*, *pheA*, *tyrB*.	Fed-batch	Glucose	8.8 g L^{-1} (36 h)	8.7%mol mol-glucose^{-1}	Koma et al. 2023
	E. coli	Overexpression of *aroG*^{fbr}, *tyrC* (cyclohexadienyl dehydrogenase, *Zymomonas mobilis*), *hpaBC*, *ARO10* (phenylpyruvate decarboxylase, *Saccharomyces cerevisiae*), and *ADH6* (alcohol dehydrogenase, *Saccharomyces cerevisiae*) along with deletions of *tyrR*, *crr*, *ptsG*, and *pheA*. Overexpression of *LAAD* (L-amino acid deaminase, *Proteus mirabilis*) for $FADH_2$ regeneration.	Fed-batch	Glycerol	9.87 g L^{-1} (35 h)	Not described	Wang et al. 2023
Salidroside	*E. coli*	Overexpression of *aroG*^{fbr}, *tyrA*^{fbr}, *aroE*^{fbr}, and *KDC4* (decarboxylase, *Pichia pastoris*) along with deletions of *ptsG*, *tyrR*, *pykA*, *pykF*, *pheA*, *feaB*,	Batch, co-culture	Glucose and xylose	6.03 g L^{-1} (129 h)	Not described	Liu et al. 2018

		manZ, and *maoB-paaY* for strain BW25113, while overexpression of *UGT85A1* (glycosyltransferase, *Arabidopsis thaliana*), *pgm*, and *galU* along with deletions of *xylA*, *ushA*, and *tyrA* for strain BL21.					
4-Hydroxymandelate	*E. coli*	Overexpression of *aroG*^{fbr}, *tyrA*^{fbr}, *aroE*, *ppsA*, *tktA*, *glk*, and *shmaS* (4-hydroxymandelate synthase, *Amycolatopsis orientalis*) and deletions of *ptsG*, *tyrR*, *pykA*, *pykF*, *pheA*, *tyrB*, *and aspC*.	Fed-batch	Glucose and xylose	15.8 g L^{-1} (60 h)	Not described	Li et al. 2016
Tyramine	*E. coli*	Overexpression of *aroF*^{fbr}, *tyrA*^{fbr}, and TyrDC (Tyr decarboxylase, *Lactobacillus brevis*).	Batch	Glucose	6.3 mM (48 h)	Not described	Koma et al. 2012a
	E. coli	Overexpression of *aroF*^{fbr}, *tyrA*^{fbr}, and *PheDC* (aromatic amino acid decarboxylase, *Enterococcus faecium*) along with deletions of *tyrA*, *pheA*, *pflB*, *ldhA*, *adhE*, and *pta*.	Batch	Glycerol	1.965 L^{-1} (72 h)	Not described	Yang et al. 2022
	C. glutamicum	Overexpression of *aroG*^{D146N} (DAHP synthase, *E. coli*) and *tdc* (Tyr decarboxylase, *Levilactobacillus brevis*) along with deletion of *ldhA*. Two chromosomal loci were substituted with *trpE*^{M1L} and *pheA*^{M1L}.	Batch	Glucose	1.9 g L^{-1} (72 h)	Not described	Poethe et al. 2024
4-Hydroxystyrene	*E. coli*	Overexpression of *PAL* (Phe ammonia-lyase, *Rhodotorula glutinis*) and *pdc* (*p*-hydroxycinnamic acid decarboxylase, *Lactobacillus plantarum*) in Phe-overproducing strain ATCC31884.	Fed-batch	Glucose	0.4 g L^{-1} (54 h)	Not described	Qi et al. 2007
4-Methoxystyrene (4-Vinylanisole)	*E. coli*	Overexpression of *aroG*^{fbr}, *tyrA*^{fbr}, *tktA*, *ppsA*, *mtn*, *luxS*, and *TAL* (tyrosine ammonia lyase, *Saccharothrix espanaensis*), *PAD* (phenolate decarboxylase, *Bacillus atrophaeus*), *AIMT1* (*O*-methyltransferase, *Pimpinella anisum*)	Batch, two-phase organic overlay culture with dodecane	Glucose	206 mg L^{-1} (36 h)	Not described	Hu et al. 2024
Salvianic acid A	*E. coli*	Overexpression of *aroG*^{D146N}, *tyrA*$^{M53I/A354V}$, *aroE*, *ppsA*, *tktA*, *glk*, *hpaBC*, and *ldhA*^{Y52A} (D-lactate dehydrogenase, *Lactobacillus pentosus*) along with deletions of *tyrR*, *ptsG*, *pykA*, *pykF* and *pheA*.	Fed-batch	Glucose	7.1 g L^{-1} (70 h)	0.47 mol mol-glucose^{-1}	Yao et al. 2013

Coumarate	*E. coli*	Overexpression of *AtPAL2* (Phe ammonia-lyase), *AtC4H*$^{L373T/G211H}$ (NADPH-cytochrome P450 reductase), and *AtATR2* (cinnamate 4-hydroxylase, *Arabidopsis thaliana*) and *ppnk* (NAD kinase, *C. glutamicum*) in Phe-overproducing strain PHE05.	Fed-batch	Glucose	3.09 g L^{-1} (63 h)	20.01 mg g-glucose^{-1}	Qiu et al. 2024
	C. glutamicum	Overexpression of *talFjCg* (Tyr ammonia-lyase, *Flavobacterium johnsoniae*) and *aroH* (DAHP synthase, *E. coli*) along with deletions of *phdA* and *pyk*. The corresponding chromosomal loci were substituted with *aroF*^{S188C}, *aroK*^{ATG}, *trpE*^{P304S}, and *pheA*^{GTG}, respectively. Constitutive expression of *aroF* (DAHP synthase, *E. coli*).	Batch	Glucose	661 mg L^{-1} (24 h)	0.017 g g-glucose^{-1}	Mutz et al. 2023
DOPA	*E. coli*	Overexpression of *galP*, *glk*, *aroG*^{fbr}, *tyrA*^{fbr}, *tktA*, *ppsA and hpaB*^{G295R}*C* along with deletions of *tyrR*, *ptsG*, *crr*, *pheA*, and *pykFA*.	Fed-batch	Glucose and glycerol (glycerol-feeding)	25.53 g L^{-1} (48 h)	Not described	Fordjour et al. 2019
Trp derivative							
Indigo and Indirubin	*E. coli*	Overexpression of FMO (flavin-containing monooxygenase, *C. glutamicum*).	Batch	Trp	685 (indigo) and 103 (indirubin) mg L^{-1} (48 h)	Not described	Ameria et al. 2015
Violacein	*E. coli*	Overexpression of *tktA*, *aroG*^{fbr}, *trpE*^{fbr}, *aroL*, *vioABCDE* (violacein synthetic enzymes, *Chromobacterium violaceum*) and deletions *trpR*, *pykF*, and *pykA*. In addition, overexpression of *cav1* and downregulation of *rfaI* (extracellular transport system using membrane vehicle).	Fed-batch	Glycerol	6.69 g L^{-1} (123.5 h)	Not described	Yang et al. 2021
	C. glutamicum	Overexpression of synthetic *vioABCDE* operon (violacein synthetic enzymes, *Chromobacterium violaceum*) in Trp-overproducing strain ATCC 21850.	Fed-batch	Glucose	5436 mg L^{-1} (115 h)	0.054 g g-glucose^{-1}	Sun et al. 2016
Serotonin	*E. coli*	Overexpression of *guaB, folE* (GTP cyclohydrolase I, *Bacillus subtilis*), *PTPS* (6-pyruvoyl-tetrahydropterin synthase, *Rattus norvegicus*) and *SPR* (sepiapterin reductase, *Rattus norvegicus*) for BH4 synthesis, *nfsB*	Fed-batch	Trp	1.68 g L^{-1} (120 h)	40.3% mol mol-Trp^{-1}	Shen et al. 2022

		and *PCD* (pterin-4α-carbinolamine dehydratase, *Pseudomonas aeruginosa*) for BH4 regeneration, *djpth* (Trp hydroxylase, *Dugesia japonica*) and *ssddc* (DOPA decarboxylase, *Sus scrofa*) for serotonin synthesis along with deletions of *pgi*, *gdhA*, and *tnaA*.					
Melatonin	*E. coli*	Overexpression of *trpH*$^{E2K/N97I/P99C}$ (Trp hydroxylase, human), *asmt*^{A258E} (acetylserotonin *O*-methyltransferase, human), *pcd* (pterin-4α-carbinolamine dehydratase), and *aanat* (aralkylamine acetyltransferase, *Streptomyces griseofuscus*) and expression of *ddc* (aromatic-amino-acid decarboxylase, Candidatus *Koribacter versatilis*) along with deletion of *trpR*, *tyrA*, *tnaA*, and *yddG*. Chromosomal mutation of *folE*^{T198I}.	Fed-batch	Trp	1538 mg L^{-1} (70 h)	Not described	Luo et al. 2020
5-Hydroxytryptophan	*E. coli*	Overexpression of *trpE*^{fbr}*DCBA*, *aroG*^{fbr}, *serA*$^{H344A/N364A}$ for Trp synthesis, *gdh* (glucose dehydrogenase, *Exiguobacterdium sibiricum*) for NAD(P)H regeneration, and *mtrA* (*Bacillus subtilis*, GTP cyclohydrolase I), *SPR* (human, sepiapterin reductase), *PTPS* (human, 6-pyruvate-tetrahydropterin synthase), *PCD* (human, pterin-4α-carbinolamine dehydratase), *DHPR* (human, dihydropteridine reductase) for BH4 synthesis and regeneration, and *TPH2*$^{E2K/N97I/P99C}$ (Trp hydroxylase deleted 145 N- and 24 C-terminuses domains, human) for hydroxylation of Trp. Mutation of *mlc* for gene overexpression by xylose.	Fed-batch	Glucose	8.58 g L^{-1} (32 h)	0.095 g g-glucose^{-1}	Zhang et al. 2022
Indole-3-acetate	*E. coli*	Overexpression of *aspC* (aminotransferase, *E. coli*), *ipdC* (indole-3-pyruvate decarboxylase, *Enterobacter cloacae*), and *iad1* (indole-3-acetate dehydrogenase, *Ustilago maydis*) along with deletion of *tnaA*.	Batch	Trp	3.0 g L^{-1} (24 h)	0.87 mol mol-Trp^{-1}	Romasi et al. 2013

E. coli	Overexpression of *ARO8* (aminotransferase, *Saccharomyces cerevisiae*), *kdc* (2-keto acid decarboxylase, *Saccharomyces cerevisiae*), *aldH* (aldehyde dehydrogenase, *E. coli*) in aromatic aldehyde accumulating strain RARE.	Batch	Glucose	0.744 g L^{-1} (24 h)	0.0372 g g-glucose^{-1}	Guo et al. 2019
E. coli	Overexpression of *iaaM* (tryptophan 2-monooxygenase, *Pseudomonas savastanoi*) and *ami1* (amidase 1, *Arabidopsis thaliana*)	Batch	Trp	7.1 g L^{-1} (42 h)	0.83 mol mol-Trp^{-1}	Wu et al. 2021
E. coli	Overexpression of the above genes and *trp* operon, *aroG*^{fbr}, *trpE*^{fbr}, *serA*^{fbr}, and *icd* (isocitrate dehydrogenase) along with deletion of *adhE*.	Batch	Glucose	0.906 g L^{-1} (84 h)	0.0453 g g-glucose^{-1}	
C. glutamicum	Overexpression of *aspC* (aminotransferase, *E. coli*), *ipdC* (indole-3-pyruvate decarboxylase, *Enterobacter cloacae*), and *iad1* (indole-3-acetate dehydrogenase, *Ustilago maydis*)	Fed-batch	Trp and glucose	7.3 g L^{-1} (62 h)	Not described	Kim et al. 2019

Advanced studies reported after 2006 are summarized.

[a)] fbr represents the gene encoding feedback-resistant enzyme.

[b)] The respective homologous genes of *E. coli* and *C. glutamicum* are as follows: *adhE*, bifunctional aldehyde-alcohol dehydrogenase; *aldH*, aldehyde dehydrogenase; *ami1*, amidase 1; *aspC*, aspartate aminotransferase; *aroA*, 5-enolpyruvylshikimate-3-phosphate synthase; *aroB*, 3-dehydroquinate synthase; *aroC*, chorismate synthase; *aroD*, 3-dehydroquinate dehydratase; *aroE*, shikimate dehydrogenase; *aroF*, 3-deoxy-D-arabinoheptulosonate-7-phosphate synthase; *aroG*, 3-deoxy-D-arabinoheptulosonate-7-phosphate synthase; *aroK*, shikimate kinase; *aroL*, shikimate kinase; *crr*, PTS system glucose-specific EIIA component; *cysE*, L-serine *O*-acetyltransferase; *feaB*, phenylacetaldehyde dehydrogenase; *folE*, GTP cyclohydrolase 1; *galP*, galactose-proton symporter; *galU*, UTP-glucose-1-phosphate uridylyltransferase; *gdhA*, glutamate dehydrogenase; *glk*; glucose kinase; *guaB*, inosine 5′ -monophosphate dehydrogenase; *hpaB*, 4-hydroxyphenylacetate 3-monooxygenase; *hpaC*, flavin reductase; *iaaM*, tryptophan 2-monooxygenase; *iad1*, indole-3-acetate dehydrogenase; *icd*, isocitrate dehydrogenase; *ipdC*, indole-3-pyruvate decarboxylase; *kdc*, 2-keto acid decarboxylase; *lacI*; DNA-binding transcriptional repressor; *ldhA*, D-lactate dehydrogenase; *luxS*, *S*-ribosylhomocysteine lyase; *manZ*, mannose-specific PTS enzyme IID component; *maoB*, synonym of *feaB*; *metA*, homoserine succinyltransferase; *mlc*, DNA-binding transcriptional repressor; *mtn*, 5-methylthioadenosine/S-adenosylhomocysteine nucleosidase; *nfsB*, dihydropteridine reductase; *paaY*, phenylacetic acid degradation protein; *pabA*, aminodeoxychorismate synthase component 2; *pckA*, phosphoenolpyruvate carboxykinase; *pflB*, pyruvate formate lyase; *pgi*, glucose-6-phosphate isomerase; *pgm*, phosphoglucomutase; *phdA*, acyl:CoA ligase; *pheA*, chorismate mutase/prephenate dehydratase; *ppsA*, phosphoenolpyruvate synthetase; *ptsG*, glucose-specific PTS enzyme IIBC component; *ptsH*, phosphocarrier protein HPr; *ptsI*, phosphoenolpyruvate-protein phosphotransferase; *pyk*, pyruvate kinase; *pykA*, pyruvate kinase; *pykF*, pyruvate kinase; *rfaI*, lipopolysaccharide glucosyltransferase (synonym of *waaO*); *serA*, D-3-phosphoglycerate dehydrogenase; *tktA*, transketolase; *trpAB*, tryptophan synthase; *trpC*, anthranilate synthase; *trpDE*, anthranilate synthase; *trpR*, Trp operon repressor; *tyrA*, chorismate mutase/prephenate dehydrogenase; *tyrB*, aromatic amino acid aminotransferase; *tyrR*, DNA-binding transcriptional dual regulator; *ushA*, 5'-nucleotidase/UDP-sugar hydrolase; *xylA*, xylose isomerase; *yahK*, aldehyde reductase; shikimate kinase; *yddG*, aromatic amino acid exporter; *ydiB*, shikimate dehydrogenase
